# Effects of Heterologous tRNA Modifications on the Production of Proteins Containing Noncanonical Amino Acids

**DOI:** 10.3390/bioengineering5010011

**Published:** 2018-02-02

**Authors:** Ana Crnković, Oscar Vargas-Rodriguez, Anna Merkuryev, Dieter Söll

**Affiliations:** 1 Department of Molecular Biophysics and Biochemistry, Yale University, New Haven, CT 06520, USA; ana.crnkovic@yale.edu (A.C.); oscar.vargas@yale.edu (O.V.-R.); 2 Department of Chemistry, Yale University, New Haven, CT 06520, USA; anna.merkuryev@yale.edu

**Keywords:** noncanonical amino acids, genetic code expansion, protein translation, tRNA, aminoacyl-tRNA synthetases, posttranscriptional modifications, phosphoserine

## Abstract

Synthesis of proteins with noncanonical amino acids (ncAAs) enables the creation of protein-based biomaterials with diverse new chemical properties that may be attractive for material science. Current methods for large-scale production of ncAA-containing proteins, frequently carried out in *Escherichia coli*, involve the use of orthogonal aminoacyl-tRNA synthetases (o-aaRSs) and tRNAs (o-tRNAs). Although o-tRNAs are designed to be orthogonal to endogenous aaRSs, their orthogonality to the components of the *E. coli* metabolism remains largely unexplored. We systematically investigated how the *E. coli* tRNA modification machinery affects the efficiency and orthogonality of o-tRNA^Sep^ used for production of proteins with the ncAA *O-*phosphoserine (Sep). The incorporation of Sep into a green fluorescent protein (GFP) in 42 *E. coli* strains carrying deletions of single tRNA modification genes identified several genes that affect the o-tRNA activity. Deletion of cysteine desulfurase (*iscS*) increased the yield of Sep-containing GFP more than eightfold, while overexpression of dimethylallyltransferase MiaA and pseudouridine synthase TruB improved the specificity of Sep incorporation. These results highlight the importance of tRNA modifications for the biosynthesis of proteins containing ncAAs, and provide a novel framework for optimization of o-tRNAs.

## 1. Introduction

Our increasing understanding of protein structure and function, together with the ease of robust recombinant protein expression systems, facilitates the design of protein-based biomaterials. These biopolymers are of great interest in the field of materials science due to their mechanical and biological properties that are, in many cases, superior to those of synthetic biomaterials. Protein-based materials have become particularly important in the development of medical devices and drug discovery because of their chemical flexibility, biocompatibility, and biodegradability [1]. A disadvantage in the design and fabrication of proteinogenic materials has been the small number of building blocks with reactive handles available through the twenty canonical amino acids. However, recent methodological advances to genetically incorporate noncanonical amino acids (ncAAs) into proteins have expanded the chemical functionalities of natural proteins and allowed the design of more sophisticated polypeptides [2]. Over 150 chemically and structurally diverse ncAAs have been genetically incorporated into proteins, which have enabled a variety of applications across different disciplines [3]. Photoactive [4], fluorinated [5], unsaturated [6], and β-amino acids [7] have been incorporated to facilitate processes such as photopatterning [8], to enhance protein stability [6], or to create proteins with unique properties [9]. However, incorporation of each ncAA requires a devoted aminoacyl-tRNA synthetase (aaRS), which first needs to be created by directed evolution [10]. This can be bypassed, in part, by genetic incorporation of the ncAA *O-*phosphoserine (Sep), whose phosphate group can be converted to a myriad of functional groups by a recently reported method [11].

Robust production of ncAA-containing proteins requires altered translational components. Primary is an engineered aminoacyl-tRNA synthetase (aaRS)•tRNA pair specific for the desired ncAA. This orthogonal translation system (OTS) must operate in a host cell being agnostic of the endogenous aaRSs and tRNAs, while interacting normally with the host translation machinery (e.g., elongation factor and ribosome, [12]). For these reasons, most OTSs are obtained from organisms of a different domain of life; thus, the OTSs used in *Escherichia coli* are usually of archaeal or eukaryotic origin [3,12]. In addition, site-specific ncAA incorporation needs codon reassignment. The major strategies are based on stop codon (UAG) suppression to minimize errors in sense codon decoding during protein synthesis. Collectively, this process is known as genetic code expansion. The current challenges are low orthogonality and polyspecificity which affect the yield and purity of the synthesized protein [13]. Lack of orthogonality may result from anticodons in orthogonal tRNAs (o-tRNAs) that are recognized by host aaRSs causing formation of the wrong aa-tRNA and leading to incorporation of an undesired amino acid [14]. Installation of antideterminants against the offending host aaRSs will prevent misacylation. However, improving OTS specificity will require a labor-intensive redesign of the orthogonal aaRS (o-aaRS) [10]. Yet, for some exotic ncAAs, engineering of other cellular components is sometimes necessary (e.g., the elongation factor Tu (EF-Tu) [15], the ribosome [7], or amino acid transporters [16]).

Although substantial progress has been made in tackling the issues described above, further improvement of OTSs will require consideration of other cellular factors that may influence ncAA translation. For instance, little is known about how the post-transcriptional modification system of the host interacts with and affects the activity of o-tRNAs. In *E. coli*, many different tRNA modifications [17] are responsible for accurate tRNA aminoacylation and codon/anticodon pairing on the ribosome. Thus, heterologous modifications of o-tRNAs may significantly influence their activity. Because most OTSs used in *E. coli* include foreign tRNAs, it is difficult to predict whether their modification pattern is similar to that of endogenous *E. coli* tRNAs. To investigate this, we employed a translation system for site-specific Sep incorporation (Sep-OTS) in *E. coli*. Using single-gene knockout strains of tRNA modification genes [18], and a green fluorescent protein (GFP) reporter system [19], we identified *E. coli* enzymes whose absence influences homogeneity of Sep-containing proteins. Unexpectedly, we found that the deletion of cysteine desulfurase gene (*iscS*) increases the total yield of GFP sixfold, while preserving the relative ratio of phosphorylated protein in the mixture. Taken together, these results underscore the essential role of tRNA modifications in genetic code expansion studies.

## 2. Materials and Methods

### 2.1. Strains and Plasmid Constructions

Oligonucleotide synthesis, DNA sequencing, and LC-MS/MS were performed by the Keck Foundation Biotechnology Resource Laboratory at Yale University. *O*-phospho-l-serine (Sep) was purchased from Sigma-Aldrich (Milwaukee, WI USA). *E. coli* TOP10 cells were used for general cloning. *E. coli* BL21(DE3), BW25113, and selected Keio knockout strains (Supplementary Table S1, [18]) were used for super-folder GFP (sfGFP) production. The reporter-containing plasmids (pET-sfGFP-sepT and all derivatives) (Supplementary Table S2) were adapted from a previously developed pET-sfGFP-pylT plasmid [19]. The plasmid pET-sfGFP-sepT-Trm5 was generated by introducing the gene encoding Trm5 from *Methanococcus maripaludis* under the *lpp* promoter and the *rrnC* terminator. A codon-optimized version of the SepRS gene was placed under an *lpp* promoter in a modified pCDF2 vector. For the experiments involving archaeal Trm5, an engineered variant of SepRS was used (SepRS9), encoded in the same manner in the pCDF2 plasmid. Plasmids pCDF-lpp.SepRS-Para.MiaA and pCDF-lpp.SepRS-Para.TruB were created by introducing *araC* and the arabinose promoter into a pCDF2 backbone (Supplementary Figure S1).

### 2.2. Growth Media

All *E. coli* strains were grown in Luria-Bertani (LB) medium, supplemented with 5 mM Sep where indicated, and sfGFP production was induced by adding 1 mM isopropyl β-d-1-thiogalactopyranoside (IPTG). MiaA and TruB expression from the corresponding pCDF vectors was induced by 1 mM arabinose.

### 2.3. SfGFP-Based Activity Assays

High-throughput sfGFP stop codon read-through assays were carried out in 96-well plates as previously described with minor modifications [20]. Briefly, *E. coli* strains were either transformed with pET-sfGFP-sepT or co-transformed with pET-sfGFP-sepT and pCDF2-SepRS. Individual colonies were grown in 1.5 mL LB medium supplemented with 50 μg/mL spectinomycin and 100 μg/mL ampicillin at 37 °C. Keio collection knockout strains were grown in 12.5 μg/mL kanamycin. Overnight cultures were diluted 1:100 in 100 µL LB medium (with or without Sep and IPTG) and transferred to a 96-well assay plate (CORNING). Growth and fluorescence (excitation wavelength 485/20 nm; emission wavelength 528/20 nm) were monitored for 24 h at 37 °C in a Synergy HT plate reader (BioTek). To quantify the amount of GFP synthesized, values obtained for relative fluorescence units (RFU) and OD_600_ in blank wells were subtracted from corresponding values collected in wells containing cells of interest. Then, the corrected fluorescence units were divided by the corrected OD_600_. The mean and standard deviations correspond to the average of at least three distinct colonies, each measured with two technical replicates.

### 2.4. SfGFP Purification and Phos-Tag Analysis

A single colony containing pET-sfGFP-2TAG-sepT(G37A) and pCDF2-SepRS or pCDF-lpp.SepRS-Para.MiaA and pCDF-lpp.SepRS-Para.TruB was inoculated into 1.5 mL LB medium and grown overnight. The overnight culture was diluted 1:200 in 25 mL LB medium with 5 mM Sep. Cells were grown to an OD_600_ of 0.5, after which the protein expression was induced. Cells were grown for 12 h after induction. Cells were lysed using BugBuster (EMD Millipore, Billerica, MA USA) and buffer A (50 mM Tris, pH 7.5, 300 mM NaCl, 10 mM imidazole, pH 7.6 and 10% (*v*/*v*) glycerol). Lysed cells were centrifuged at 11,000× *g* for 30 min at 4 °C, and the supernatant was loaded on a pre-equilibrated Ni-NA slurry (200 µL). The resin was washed with buffers A and B (same as A but with 35 mM imidazole). Finally, the protein was eluted with buffer C (300 mM imidazole, pH 7.6, 200 mM NaCl, 5% (*v*/*v*) glycerol). Eluted protein was then concentrated to ~A_280_ = 1 and stored at −20 °C.

For the Phos-tag^TM^ mobility shift and Western blot analysis of sfGFP, 2 µg of isolated sfGFP was loaded onto a 10% or 12% polyacrylamide gel containing 50 µM Mn^2+^-Phos-tag^TM^ acrylamide (Wako Pure Chemical Industries, Ltd., Osaka, Japan). After electrophoresis, the gel was washed in transfer buffer with 1 mM EDTA to remove the Mn^2+^ ions and then in an EDTA-free transfer buffer (25 mM Tris, 191 mM Gly, 20% (*v*/*v*) ethanol). PVDF membrane was soaked in methanol and then equilibrated in the transfer buffer. Transfer was executed at 65 mA for 30 min. The membrane was blotted with 1:1000 anti-GFP (rabbit IgG fraction, conjugated to horseradish peroxidase, Life Technologies, Grand Island, NY USA). The chemiluminescent signal was detected and captured on a ChemiDoc MP camera (Bio-Rad, Hercules, CA, USA). Recorded signals were quantified by ImageJ (developer Wayne Rasband, Research Services Branch of NIH, Research Triangle Park, NC, USA) [21].

## 3. Results and Discussion

### 3.1. Implications of Post-Transcriptional Modifications in Orthogonal tRNAs

Because of their pivotal role in translation of the genetic code, tRNAs are tightly regulated through an intricate metabolic cycle which includes a myriad of post-transcriptional chemical modifications across the tRNA structure. Over 100 currently identified modifications perform versatile tasks in tRNA processing, stability, and functionality [17,22,23]. tRNAs are more comprehensively modified in the anticodon region, especially at positions 34 (first base of the anticodon) and 37 (base following the anticodon) [24]. These particular modifications are critical for faithful translation of the genetic code as they ensure proper amino acid pairing with cognate tRNAs and correct matching of the codon/anticodon at the ribosome [25,26]. However, bacterial, archaeal, and eukaryal enzymes devoted to their formation sometimes differ and tRNAs from different kingdoms may bear different nucleotide determinants (e.g., [27]). Despite their essential role in protein synthesis, and the fact that o-tRNAs might be undermodified or improperly modified within the host, tRNA modifications have been mostly overlooked in the development or optimization of OTSs.

**Figure 1:**
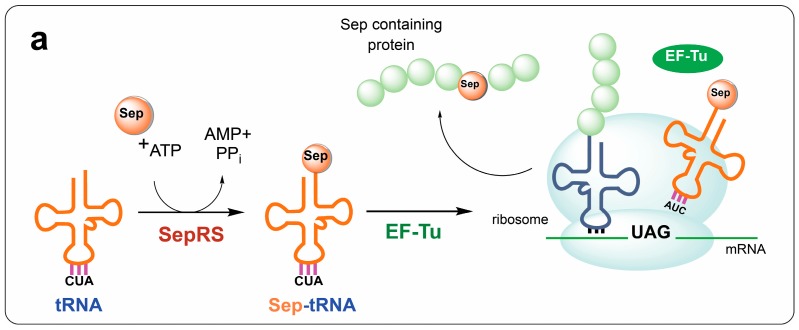

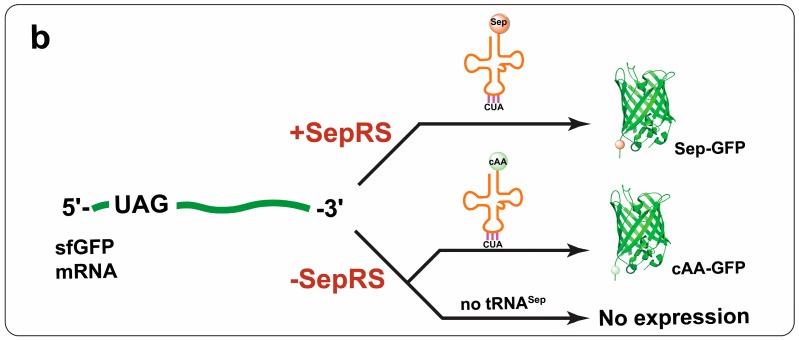
(**a**) Schematic representation of stop codon suppression with noncanonical amino acids (ncAAs). An orthogonal aminoacyl-tRNA synthetase (aaRS) (represented by SepRS) aminoacylates an orthogonal suppressor tRNA (anticodon CUA, shown in orange) with a noncognate amino acid (Sep). Sep-tRNA^Sep^_CUA_ is delivered to the ribosome by the modified version of the host elongation factor EF-Tu. At the ribosome, the Sep-tRNA^Sep^_CUA_ decodes an internal stop codon (UAG) and the ncAA is incorporated in the nascent protein. (**b**) Schematic depiction of a stop codon read-through assay using super-folder green fluorescent protein (sfGFP)-2TAG. A stop codon containing mRNA can be translated only in the presence of suppressor tRNA (here, tRNA^Sep^_CUA_). In the presence of SepRS, tRNA^Sep^_CUA_ is aminoacylated with phosphoserine, which is subsequently incorporated into sfGFP. In the absence of SepRS, an endogenous aaRS may misacylate tRNA^Sep^_CUA_ with a canonical amino acid, which then becomes incorporated into sfGFP.

Here we explored the influence of *E. coli*-specific post-transcriptional modifications on the performance of Sep-OTS. tRNA^Sep^_CUA_ was originally created by introducing three base substitutions in the archaeal *Methanocaldococcus jannaschii* tRNA^Cys^ [15]. *M. maripaludis* phosphoseryl-tRNA synthetase (SepRS) aminoacylates tRNA^Sep^_CUA_ with phosphoserine, thereby enabling site-specific incorporation of Sep in response to a UAG stop codon (Figure 1a). The Sep-OTS has been of particular interest since it simplifies the preparation of Sep-containing proteins and enables precise placement of Sep within a protein. Naturally present in proteins as a reversible post-translational modification, Sep is fundamental in the regulation of protein activity in all organisms. While Sep-OTS has already aided mechanistic studies of serine phosphorylation/dephosphorylation (e.g., [28]), the overall efficiency and specificity of the system has presented complications that compromise sample yields and purity. To overcome these issues, most efforts so far have focused on improving aminoacylation efficiency by engineering of tRNA^Sep^ [29,30], SepRS [30,31], and elongation factor Tu (EF-Tu, [15,31]). In the case of tRNA^Sep^, optimization has involved the evolution of more efficient tRNA^Sep^ mutants [30] and/or the improvement of tRNA expression levels [29], with no consideration to the potential role of heterologous modifications on tRNA^Sep^.

**Figure 2:**
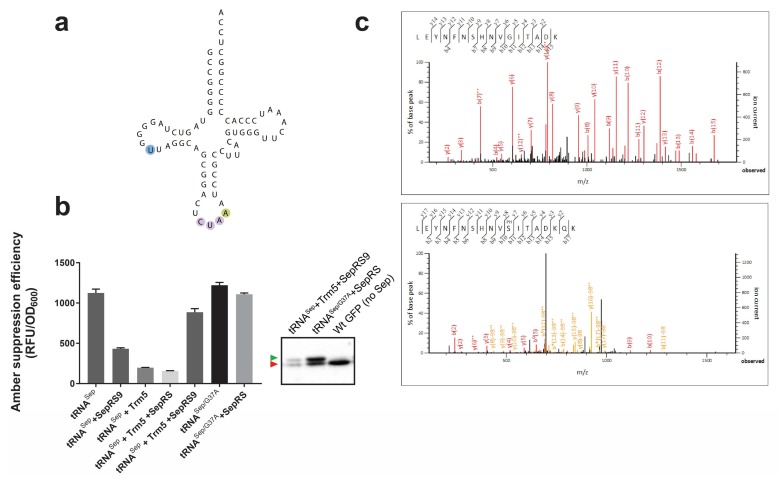
(**a**) Secondary structure of tRNA^Sep/G37A^. Anticodon bases are highlighted and the G37A mutation is indicated. (**b**) The effect of *M. maripaludis* Trm5 on the stop codon read-through with original tRNA^Sep^ and tRNA^Sep/G37A^ in the presence or absence of wild-type SepRS or SepRS9 variant. sfGFP-2TAG expression was monitored by fluorescence in a BL21(DE3) strain grown in Luria-Bertani (LB) medium supplemented with 5 mM Sep. Responses were taken at 20 h. The amber suppression efficiency is measured relative to the cell density (OD_600_). Phos-tag^TM^ mobility shift analysis of GFP-2TAG isolated from the strains harboring tRNA^Sep^, SepRS9, and Trm5 and tRNA^Sep/G37A^ variant and wild-type SepRS. OTS components are indicated above the wells. Proteins were detected by Western blot using anti-GFP antibodies. The green arrow indicates the position of the Sep-containing GFP, while the red one points to the position of cAA-containing GFP. (**c**) Endogenous GlyRS likely charges MjtRNA^Sep^ with Gly. LC-MS/MS analyses of sfGFP-151TAG demonstrate that in the presence of Sep-OTS (tRNA^Sep^_CUA_^G37A^ system), both Sep and Gly incorporation occur. The tandem mass spectrum of the peptides (residues 141–156) LEYNFNSHNVGITADK (ion score 116.5) and LEYNFNSHNVSepITADK (ion score 52.9) from purified sfGFP with one amber codon at position 151 from *E. coli* BL21 (DE3), using the tRNA^Sep^_CUA_^G37A^-containing Sep-OTS. Cells were grown without additional Sep in the medium.

To gain insights into whether modifications influence o-tRNA activity in the host organism, we began our investigation using the first generation of tRNA^Sep^, which contains G at position 37 (Figure 2a). In *M. jannaschii*, the parental tRNA^Cys^ is methylated at G37 by the methyltransferase Trm5 [27]. Because this modification substantially increases aminoacylation efficiency by SepRS [32], G37 methylation of tRNA^Sep^ in *E. coli* might increase the overall efficiency of the Sep-OTS. However, it is unknown whether the essential *E. coli* methyltransferase TrmD—an evolutionarily divergent homolog of Trm5—can catalyze the methylation of tRNA^Sep^ G37 [18]. We were unable to assess the influence of TrmD on tRNA^Sep^, as the *trmD* deletion strain is not part of the Keio collection. Therefore, to test whether methylation of tRNA^Sep^ G37 can increase aminoacylation by SepRS, and, consequently, Sep incorporation, we co-expressed archaeal Trm5 with tRNA^Sep^ in *E. coli.* We tested this system in the absence and presence of a SepRS mutant (SepRS9) that was previously engineered to improve the aminoacylation of tRNA^Sep^ by altering its anticodon binding domain [31]. The effect of Trm5 on tRNA^Sep^ was monitored by using a super-folder GFP (sfGFP) reporter gene [33] with an amber UAG codon replacing a Ser codon at position 2 (sfGFP-2TAG, [19]). In this assay, suppression (read-through) of the amber stop codon by tRNA^Sep^ leads to synthesis of sfGFP (Figure 1b). Using this platform, we found that the orthogonality of tRNA^Sep^ in the absence of SepRS appears to be compromised by host aaRSs (previously, Gln incorporation has been reported, [15]). However, in the presence of SepRS9, sfGFP synthesis was reduced, which is the result of tRNA^Sep^ sequestration by SepRS that prevents misacylation of tRNA^Sep^ by host aaRSs and reduces read-through. Interestingly, co-expression of Trm5 improved tRNA^Sep^ orthogonality almost sixfold. This result suggests that methylation of G37 can significantly prevent aminoacylation of tRNA^Sep^ by *E. coli* aaRSs (Figure 2b). Low sfGFP yields in cells co-expressing Trm5 and wild-type SepRS suggest that the G37 methylation does not improve the aminoacylation activity of wild-type SepRS for tRNA^Sep^ (Figure 2b). On the other hand, expression of SepRS9 with Trm5 increased the GFP yields approximately twofold, indicating that the anticodon binding domain of SepRS9 is more accommodating for the CUA anticodon with the adjacent G37 methyl group. However, the Phos-tag^TM^ analysis indicates that a significant amount of misincorporation still occurs in the presence of Trm5 and SepRS9 (Figure 2b).

Because OTS optimization entails establishing a platform that maintains the o-aaRS aminoacylation efficiency, retains or increases o-tRNA orthogonality, and maximizes EF-Tu acceptance and o-tRNA decoding capacity, we decided to test a tRNA^Sep^ variant with a G37-to-A mutation (tRNA^Sep/G37A^) that was previously shown to enhance the suppression activity of the tRNA [29]. Because G37 [34] and its methylated counterpart [32] act as determinants for SepRS aminoacylation, the success of o-tRNA^Sep/G37A^ most likely originates from its improved decoding ability [26]. In fact, adenosine at position 37 is common in strong natural and artificial UAG suppressors [3,35], suggesting that A37 improves the tRNA suppression activity. It is well known that the stabilizing modifications such as ct^6^Aand ms^2^i^6^A accompany weak A1-U36 or U1-A36 codon–anticodon base pairs in *E. coli* (the same base pairing pattern occurs in the decoding of amber stop codons). Indeed, co-expression of SepRS and tRNA^Sep/G37A^ yielded a similar suppression efficiency as that of tRNA^Sep/G37A^ alone, and a 2.5× increase in comparison to G37-containing tRNA^Sep^. Although G73A did not improve tRNA orthogonality, it improved the suppression efficiency without decreasing the percentage of specific Sep incorporation (Figure 2b). In addition to Phos-tag^TM^ analysis, we confirmed Sep incorporation in a purified recombinant sfGFP using tandem mass spectrometry (LC-MS/MS) (Figure 2c). In addition to Sep, Gly was detected. This can result from misacylation of tRNA^Sep/G37A^, which shares sequence elements with *E. coli* tRNA^Gly^, with Gly by *E. coli* glycyl-tRNA synthetase [34,36].

### 3.2. Sep-OTS Activity in the Absence of Individual Post-transcriptional Modification Enzymes

In *E. coli*, an archaeal o-tRNA can be challenged by endogenous tRNA modification enzymes that (a) may introduce bacteria-specific modifications (only half of archaeal modifications are found in bacterial milieu) and (b) can act on the o-tRNA in a sequence-specific manner characteristic of bacterial tRNAs [23,37]. On the other hand, archaeal o-tRNAs can also be challenged by the absence of native archaeal modifications. For example, specific modifications that contribute to tRNA folding and stabilization might be missing in bacteria [38,39].

To determine if heterologous tRNA modifications play a role in the activity of Sep-OTS in *E. coli*, we used the sfGFP2-TAG reporter assay in a series of *E. coli* single-gene knockout strains from Keio collection [18]. These strains are suitable for stop codon read-through assays, because they do not possess genomic copies of tRNA suppressors, and, as indicated by the sfGFP-2TAG synthesis in tRNA^Sep/G37A^ absence (Supplementary Figure S2), show very low levels (<1%) of near cognate suppression. Therefore, the observed sfGFP-2TAG production is exclusively based on the activity of tRNA^Sep/G37A^. We tested 42 strains in which a single gene encoding a nonessential enzyme involved in post-transcriptional tRNA modification was deleted (a full list of strains is given in Supplementary Table S1). Although only a few of these enzymes operate independently, we decided to investigate all enzymes participating in the biosynthesis of post-transcriptional modifications because of their mutual interactions and their connection to other metabolic processes [40,41,42].

**Figure 3:**
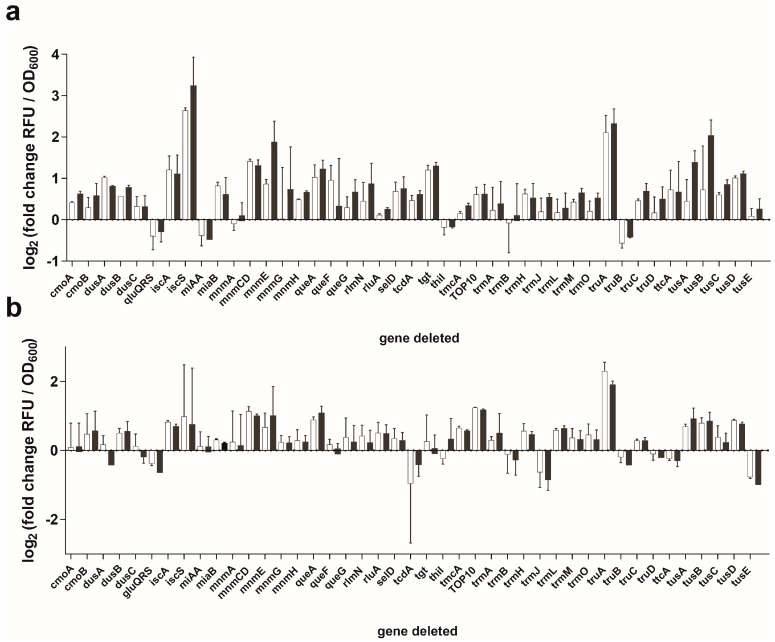
Stop codon read-through assays with sfGFP-2TAG in 42 selected Keio collection strains with full Sep-OTS (**a**) and with suppressor tRNA^Sep^_CUA_^G37A^ only (**b**). First column (white) corresponds to the signal recorded in the media without, and the second (black) with 5 mM Sep. Fold change values were derived from 4–8 (**a**) and 3–6 biological replicates (**b**).

We observed significant variations in the efficiency of the Sep-OTS in the absence of particular modification enzymes (Figure 3a, Supplementary Figures S3 and S4, Tables S3 and S4). For example, in the strains lacking GluQRS, MiaA, ThiI, or TruB, sfGFP expression was decreased. Enzymes MiaA, ThiI, and TruB are involved in the i^6^A37, s^4^U8, and Ψ55 modifications, respectively, suggesting that these modifications may be important for the activity of the Sep-OTS. How the absence of GluQRS impairs sfGFP synthesis is unclear since this enzyme operates on U34, which is missing in tRNA^Sep/G37A^ (Supplementary Table S1). Synthesis of wild-type sfGFP in Δ*thiI* and Δ*truB* strains demonstrates that in these strains the overall translation is somewhat diminished (Supplementary Figure S5 and Table S5), thereby indicating that their absence might affect the activities of endogenous tRNAs, not only tRNA^Sep/G37A^. In contrast, the yield of WT sfGFP in the Δ*miaA* strain is comparable to the yield in the wild-type BW25113 strain (Supplementary Figure S5 and Table S5), implying that the lack of i^6^A37 modification directly affects tRNA^Sep/G37A^ (and therefore Sep-OTS).

### 3.3. Effects of Post-transcriptional Modifications on the Orthogonality of tRNA^Sep/G37A^

We also analyzed the role of modifications on the orthogonality of tRNA^Sep/G37A^ by expressing this tRNA in the absence of SepRS (Figure 3b, Supplementary Figure S4). In this context, a decrease in sfGFP expression signal can be interpreted as an increase in tRNA^Sep/G37A^ orthogonality, which may be related to the absence of modifications that improve mischarging of tRNA^Sep/G37A^ by endogenous aaRSs. Mischarging with canonical amino acids (cAAs) results in the cAA-tRNA^Sep/G37A^ formation which can seemingly increase the sfGFP-2TAG synthesis, mask the true Sep incorporation efficacy, and reduce Sep incorporation by competing for the binding to the UAG codons (discussed in Section 3.4 and Section 3.5).

Some of the strains allow for minor improvements in tRNA^Sep/G37A^ orthogonality (1.4–1.8-fold) according to the mean decrease in sfGFP synthesis. Corresponding decreases were recorded in strains lacking GluQRS, TrmJ, and TusE (Figure 3b, Supplementary Table S4). Strains lacking DusA, TcdA, TruB, TtcA offer a marginal improvement of 1.2–1.4-fold. Among these enzymes, DusA, TcdA, TrmJ, TruB, and TtcA can theoretically operate on tRNA^Sep/G37A^ (Supplementary Table S1). TrmJ and TtcA modify base C32, thereby influencing the conformation of the anticodon stem-loop. The corresponding modifications Cm and s^2^C reduce the RNA conformational flexibility, thereby making this tRNA part rigid [26]. Because anticodon stem-loop modifications directly influence its conformation, the lack of these modifications may result in mirrored effects in screens with or without SepRS; in other words, the lack of modifications placed in the tRNA anticodon will, most likely, reflect the changes in the tRNAs’ decoding capacity. DusA catalyzes dihydrouridine formation on U20, which influences D-loop flexibility and tRNA folding [22]. TcdA acts on premodified base A37, which is not likely to form in the context of tRNA^Sep/G37A^ [43]. Because wild-type GFP translation is decreased in both Δ*tcdA* and Δ*ttcA*, these enzymes may affect overall translation (Supplementary Figure S5 and Table S5), whereas DusA and TrmJ may actually introduce modifications in tRNA^Sep/G37A^ that influence its orthogonality. In contrast, deletion of *truB* decreases sfGFP-2TAG synthesis in the absence and presence of SepRS; thus, it is likely that the corresponding modification influences tRNA^Sep/G37A^ stability overall, and not the interaction of tRNA^Sep/G37A^ with endogenous aaRSs.

The highest increase in sfGFP synthesis occurs in *E. coli* strains lacking IscS, MnmCD, MnmE, TGT, TruA, TusA, or TusB enzymes (Figure 3a, Supplementary Table S3). However, this effect is accompanied by a decrease in orthogonality (Figure 3b, Supplementary Table S4). In comparison with the wild-type strain, a significant decrease in orthogonality also occurs in the Δ*queA* strain. MnmCD, MnmE, QueA, and TGT should not act on tRNA^Sep/G37A^, as this tRNA does not possess the necessary nucleotide sequence for their recognition (Supplementary Table S1). It is possible that the lack of modifications on the *E. coli* tRNAs reduces the affinity of their corresponding aaRSs, resulting in improved misacylation of tRNA^Sep/G37A^. IscS, TusA, and TusB participate in the sulfur transfer required for the biosynthesis of sulfur-containing post-transcriptional modifications [44] and have no tRNA substrates as such. Curiously, sfGFP-2TAG synthesis is approximately twofold higher in *E. coli* TOP10 cells containing tRNA^Sep/G37A^, indicating that the BW25113 genetic background might be more appropriate for Sep-OTS-assisted stop codon suppression and production of Sep-containing proteins.

### 3.4. Deletion of iscS Leads to a Significant Increase of Sep-Containing sfGFP

Efficiency of site-specific incorporation of ncAAs via nonsense suppression depends on the catalytic prowess of the OTS [3], the availability of ncAA (depending on the ncAA intake and its participation in the cellular metabolism, [45]), the suitability of ncAA-o-tRNA for EF-Tu binding [12], and the capacity of ncAA-o-tRNA to decode the stop codon of interest [26] and outcompete the release factor for binding to the same site [46]. Because each of these processes is usually less efficient than for endogenous translation systems, yields of ncAA-containing proteins are typically low. Prompted by the fact that some of the deletion strains tested in this study show markedly increased yields of GFP-2TAG (Figure 3a), we decided to quantitatively explore the efficiency of Sep incorporation in these strains. We isolated GFP-2TAG synthesized in the presence of Sep-OTS in these select strains and analyzed the level of Sep incorporation by Phos-tag^TM^ technology [47].

**Figure 4:**
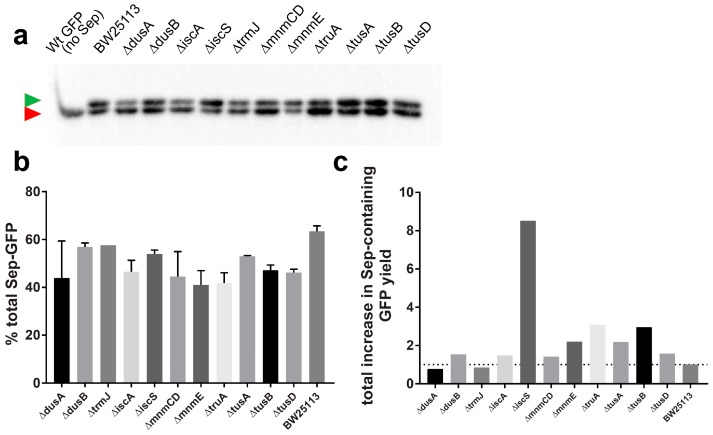
Some of the deletion strains showing increased GFP-2TAG synthesis while preserving the relative Sep-GFP to cAA-GFP ratio. (**a**) Phos-tag^TM^ mobility shift analysis of GFP-2TAG isolated from the corresponding knockout strains. Strain names are indicated above the wells. Proteins were analyzed by the Phos-tag method and detected by Western blot using anti-GFP antibodies. The green arrow shows the position of the Sep-containing GFP, while the red one points to the position of nonphosphorylated, cAA-containing GFP. Wild-type GFP controls are also shown. (**b**) Quantification of the shifted bands allows the estimation of Sep incorporation efficiency. Percentages of Sep-modified GFP in the corresponding isolate are shown. Values are presented as mean ± S.D. derived from two biological replicates. (**c**) Mean increase in total GFP-2TAG synthesis (Figure 3, Supplementary Figure S3 and Table S3) multiplied by the mean percentage of Sep-containing GFP in the matching strain shows the factual increase in Sep-GFP production.

We chose strains with medium (Δ*dusA*, Δ*dusB*, Δ*iscA*, *ΔtrmJ*, and Δ*tusD*) and high (Δ*iscS*, Δ*mnmCD*, Δ*mnmE*, Δ*truA*, Δ*tusA*, and Δ*tusB*) increases in GFP-2TAG synthesis relative to the wild-type (parental BW25113) strain. GFP isolated from these strains was subjected to Phos-tag^TM^ analysis—a mobility shift assay using an alkoxide-bridged dinuclear metal complex that can be employed to monitor stoichiometric incorporation of Sep [47]. Because phosphoserine residues form complexes with Mn^2+^-ions immobilized within the gel via chelating agent Phos-tag™, the fraction with Sep-containing GFP is decelerated in the gel and separates from the cAA-containing GFP. Using anti-GFP antibodies, these fractions can be quantified and relative amounts of Sep-GFP in the total mixture calculated (Figure 4a,b).

The result of Phos-tag^TM^ analysis is shown in Figure 4a. Densitometric quantification of the slower, Sep-containing fraction shows that in the wild-type strain, Sep-GFP accounts for 65% of the total protein. This ratio is preserved in Δ*dusB*, Δ*iscS,* Δ*trmJ*, and Δ*tusA* strains (57%, 54%, 58%, and 53% of Sep-GFP, respectively). In strains Δ*dusA*, Δ*mnmCD*, Δ*mnmE*, and Δ*truA*, it is considerably lower (33%, 37%, 37%, and 39%, respectively). The mean increase in GFP production, as monitored by the fluorescence (Figure 3a), multiplied by the fraction of Sep-containing GFP, as determined by Phos-tag^TM^ analysis (Figure 4b), allows us to establish factual increases in Sep-GFP production (Figure 4c). These values demonstrate that deletion strains lacking TruA, TusB, and MnmE, while showing more than fourfold increase in total GFP synthesis, provide a less than threefold improvement in Sep-GFP production. While this is still an improvement, Δ*dusA* and Δ*trmJ*—strains with moderate (less than twofold) increases in GFP-2TAG production (Figure 3a, Supplementary Figure S3 and Table S3)—actually produce less Sep-GFP than the wild-type (Figure 4c). In conclusion, only the *iscS* deletion strain offers a substantial improvement in Sep-GFP synthesis (Figure 4c).

### 3.5. Overexpression of MiaA and TruB Leads to a Higher Purity of Sep-Containing sfGFP

The analysis of the Sep-OTS-facilitated production of GFP-2TAG in the deletion strains (Figure 3a, Supplementary Table S3) suggested that the modifications i^6^A37, s^4^U8, and Ψ55 influence Sep-OTS performance. Because natural tRNA suppressors lacking i^6^A are significantly less active [35,48], we decided to test whether overexpression of dimethylallyltransferase MiaA can increase production of Sep-GFP. The i^6^A37 modification in the anticodon loop prevents unwanted hydrogen bond formation between the C32 and A38, as well as the A37 and U33, thereby ensuring productive conformation of this element [26]. The A36-A37-A38 motif (present in tRNA^Sep/G37A^, Figure 2a) serves as an identity element for the *E. coli* MiaA enzyme [49]. In addition to MiaA, we examined pseudouridine synthase TruB, which not only catalyzes pseudouridine formation but also acts as a general RNA chaperone [50]. We reasoned that the overexpression of MiaA and TruB may increase the fraction of tRNA^Sep/G37A^ with i^6^A37 and Ψ55, respectively, while TruB may also facilitate folding of this o-tRNA.

Genes encoding MiaA and TruB were separately introduced into a pCDF-derived plasmid containing a copy of the *SepRS* gene. We decided to place the *miaA* and *truB* genes under the control of the arabinose promoter to enable tight control of their expression, while *SepRS* expression was driven by the constitutive *lpp* promoter. This experimental design allows us to compare Sep-OTS assisted sfGFP-2TAG production with natural and artificially increased levels of MiaA and TruB.

**Figure 5:**
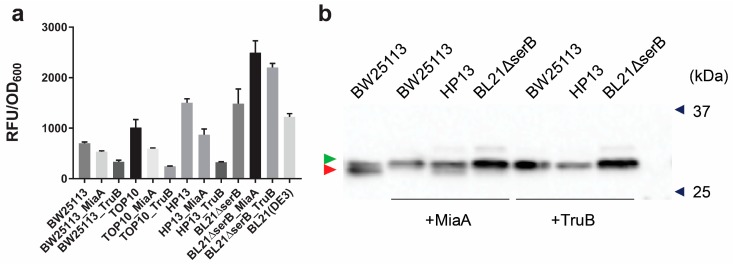
Overexpression of MiaA and TruB does not improve total yields of sfGFP-2TAG, but improves specific Sep incorporation markedly. (**a**) Stop codon read-through facilitated by Sep-OTS in the presence of overexpressed MiaA and TruB. Assay was executed in the strains BW25113 and TOP10, as well as the *serB* deletion strains BL21Δ*serB* and HP13. The values represent the mean ± S.D. derived from 4–8 biological replicates. (**b**) Phos-tag^TM^ mobility shift analysis of GFP-2TAG isolated from the corresponding strains (marked above the membrane). Overexpression of MiaA and TruB is indicated. Deleted genes are denoted above the wells, except for the parental BW25113 strain. Proteins were detected by Western blot using anti-GFP antibodies. The green arrow shows the position of the Sep-containing fraction, while the red one points to the position of nonphosphorylated, wild-type GFP. Control fraction containing GFP isolated from the BW25113 strain is also shown.

Contrary to our expectations, the total Sep-OTS assisted sfGFP-2TAG synthesis decreases upon stimulation of MiaA and TruB expression (Figure 5a). In BW25113, the mean fluorescence signal of the sfGFP-2TAG reporter decreases 1.3-fold in the case of MiaA and 2.1-fold for TruB overexpression relative to the cells expressing these enzymes solely from the chromosomal loci. However, homogeneity of Sep incorporation significantly improves upon overexpression of either MiaA or TruB, with Sep-GFP yields amounting to 81% and 88%, respectively, as analyzed by Phos-tag^TM^ (Figure 5b).

Sep is naturally synthesized in *E. coli* as part of the Ser metabolic pathway. Because Sep is effectively converted to Ser by the phosphoserine phosphatase SerB, its cellular concentration is low [45]. Thus, Sep-OTS-assisted read-through increases with Sep supplementation (Figure 3, Figure 4 , and Figures S2–S4). Alternatively, the intracellular levels of Sep can be increased by deleting the *serB* gene [15,45]. However, in the *E. coli* strains HP13 (a TOP10 derivative) and BL21Δ*serB*, which lack the *serB* gene, the stimulation of Sep-OTS-aided sfGFP-2TAG read-through is markedly different (Figure 5a,b). This may be explained by the differences in metabolic fluxes between K12 and BL21 strains [51]. Compared with its parental TOP10 strain, HP13 allows a marginal (less than 1.5-fold) improvement of sfGFP-2TAG synthesis with MiaA overexpression. In the case of TruB overexpression, sfGFP-2TAG levels are comparably low in both cell strains. In terms of Sep-GFP homogeneity, the high purity of Sep-GFP isolated from the HP13-TruB overexpressing strain is preserved (94%), while purity decreases to 42% when MiaA is overexpressed in HP13 (Figure 5 and Figure S6). In the BL21Δ*serB* and wild-type BW25113 strains, sfGFP-2TAG synthesis significantly improved when either MiaA or TruB were overexpressed (4.6- and 4.1-fold improvement, respectively, Figure S6).

Based on the Phos-tag^TM^ analysis of GFP-2TAG isolated from the wild-type BW25113 strain, a considerable fraction of tRNA^Sep/G37A^ is misacylated by *E. coli* aaRSs, which prevents the synthesis of homogenous protein samples. While orthogonality tests can estimate a tRNA’s apparent orthogonality, in the absence of the cognate o-aaRS, the pool of deacylated o-tRNA increases, allowing it to compete for binding with endogenous aaRSs more easily. This phenomenon is exemplified by the *iscS* deletion strain, where apparent orthogonality decreases approximately twofold in the stop codon read-through assay, but analysis of the relative amounts of Sep-GFP in the mixture revealed that the relative misacylation is comparable to what is occurring in the wild-type BW25113 strain. While methods to accurately quantify ncAA incorporation in proteins are costly and less accessible, they are necessary in establishing the strength and efficiency of a particular OTS [52].

## 4. Conclusions

The efficiency of Sep-OTS—a translation system designed to synthesize proteins with Sep—was evaluated in a series of *E. coli* strains with deletions of single genes responsible for nucleotide modifications of tRNAs. Several *E. coli* enzymes were identified that positively affected the synthesis of a GFP reporter variant containing Sep. An *iscS* deletion strain enables more than eightfold higher production of this variant, while overexpression of the MiaA and TruB enzymes significantly improve the specificity of the Sep incorporation. This work provides new evidence highlighting the role of tRNA modification in efficient translation. This will help the design and construction of better o-tRNAs.

## Supplementary Materials

The following are available online at http://www.mdpi.com/2306-5354/5/1/11/s1, Figure S1: Plasmid maps used in this study. Figure S2: Levels of near cognate suppression in selected Keio deletion strains. Figure S3: Levels of amber codon suppression efficiency in the selected Keio deletion in presence of Sep-OTS. Figure S4: Levels of amber codon suppression efficiency in the selected Keio deletion strains in presence of nonsense suppressor tRNA^Sep^_CUA_^G37A^. Figure S5: Levels of wild-type sfGFP synthesis in the tested Keio collection strains. Figure S6: Quantification of the shifted bands corresponding to Sep-GFP yields observed in presence of increased amounts of MiaA and TruB. Table S1: List of Keio knockout strains employed in this study. Table S2: Plasmids used in this study. Table S3: Statistical analysis of the sfGFP-2TAG synthesis in the Keio deletion strains in presence of Sep-OTS. Table S4: Statistical analysis of the sfGFP-2TAG synthesis in the Keio deletion strains in presence of tRNA^Sep/G37A^. Table S5: Statistical analysis of the sfGFP synthesis in the Keio deletion strains.

Supplementary File 1
