# Supplementary material for: Effects of Heterologous tRNA Modifications on the Production of Proteins Containing Noncanonical Amino Acids
Source: Bioengineering (Basel). 2018 Feb 2;5(1):11. doi: 10.3390/bioengineering5010011 (PMC5874877; doi:10.3390/bioengineering5010011)
Supplement: Supplementary File 1 [file bioengineering-05-00011-s001.pdf]

Supplementary material for Heterologous tRNA modifications influence o-tRNA orthogonality and OTS efficiency by Ana Crnković, Oscar Vargas-Rodriguez, Anna Merkuryev and Dieter Söll

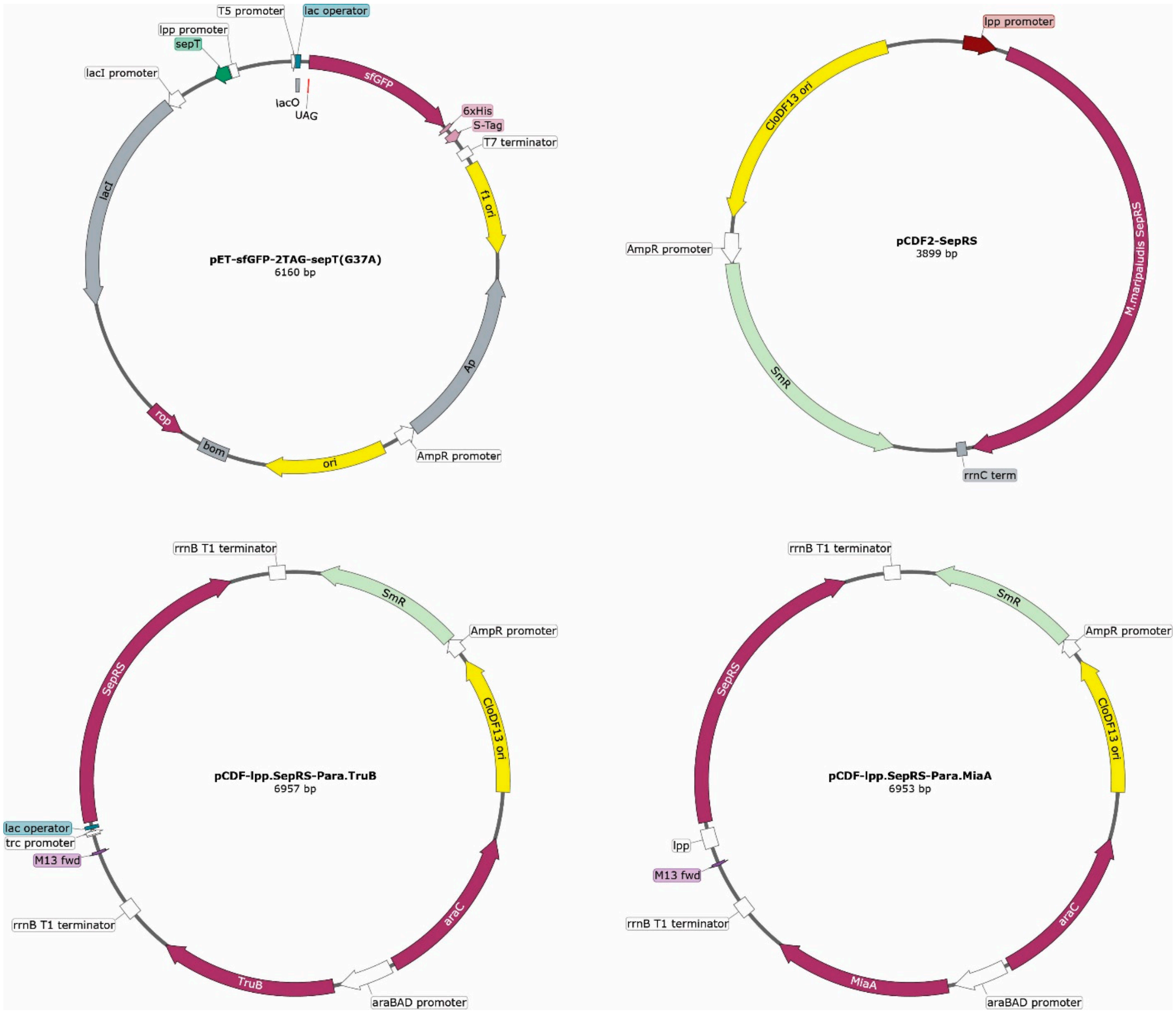

Supplementary Figure S1. Maps of plasmids used in this study.

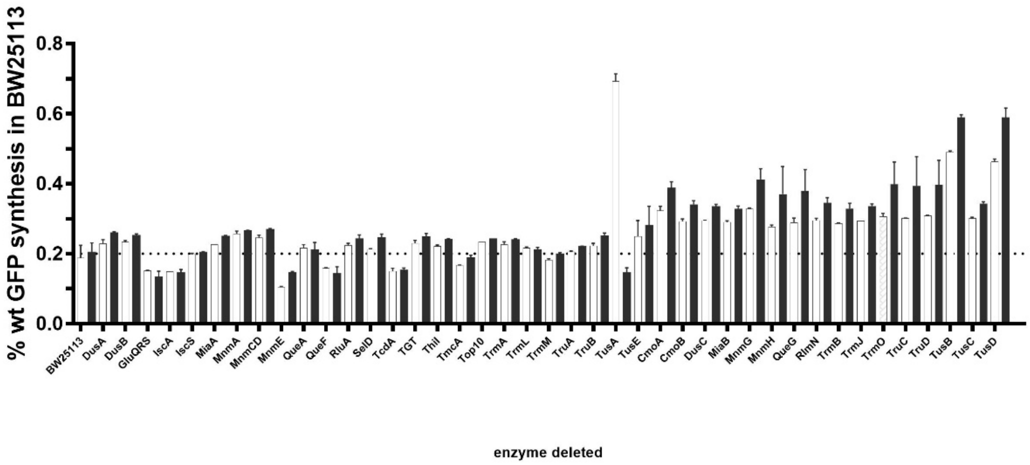

Supplementary Figure S2. Levels of near cognate suppression (measured as sfGFP-2TAG synthesis in the absence of $tRNA^{Sep}$) in the investigated Keio deletion strains are not markedly higher than those of commonly employed strains. The levels of suppression are expressed relative to the mean wild-type GFP fluorescence obtained from the parental BW25113 strain. Cells were grown in LB medium without supplementation (empty columns) or with 5 mM Sep added (black columns). Values are mean +/- S.D., N=3.

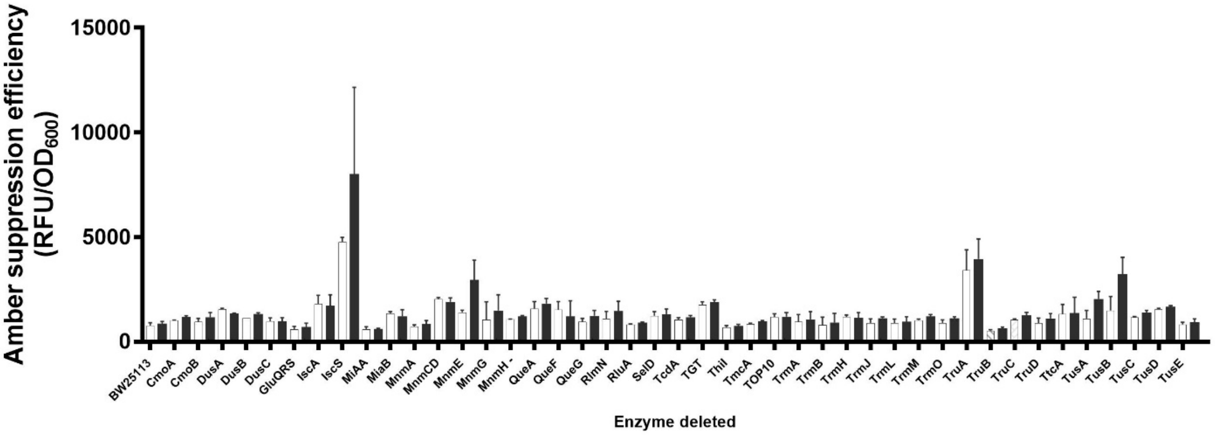

Supplementary Figure S3. Levels of amber codon suppression efficiency (measured as sfGFP-2TAG synthesis) in the selected Keio deletion strains in presence of Sep-OTS. Cells were grown in LB medium without supplementation (empty columns) or with 5 mM Sep added (black columns). Values are mean +/- S.D., N=4-8.

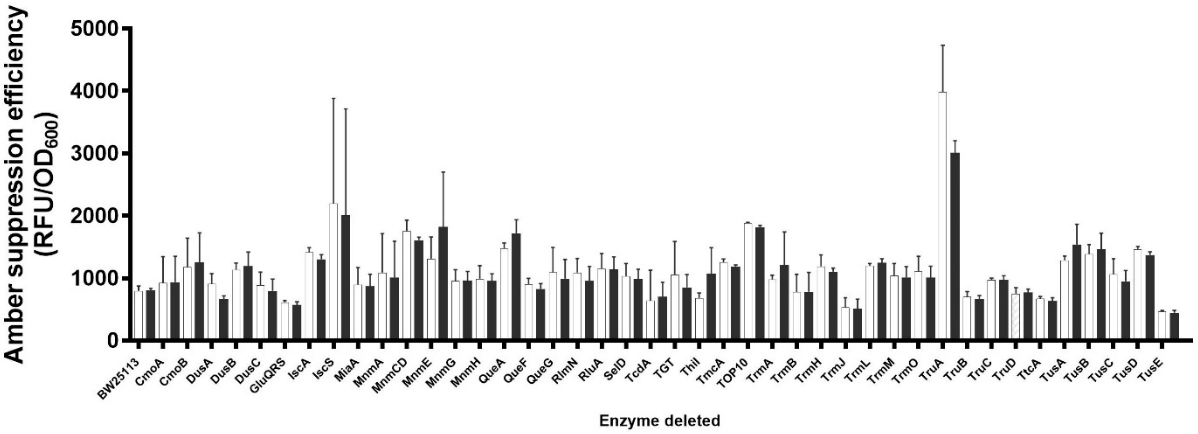

Supplementary Figure S4. Levels of amber codon suppression efficiency (measured as sfGFP-2TAG synthesis) in the selected Keio deletion in presence of nonsense suppressor $tRNA^{Sep}_{CUA}{}^{G37A}$. Cells were grown in LB medium without supplementation (empty columns) or with 5 mM Sep added (black columns). Values are mean +/- S.D., N=3-6.

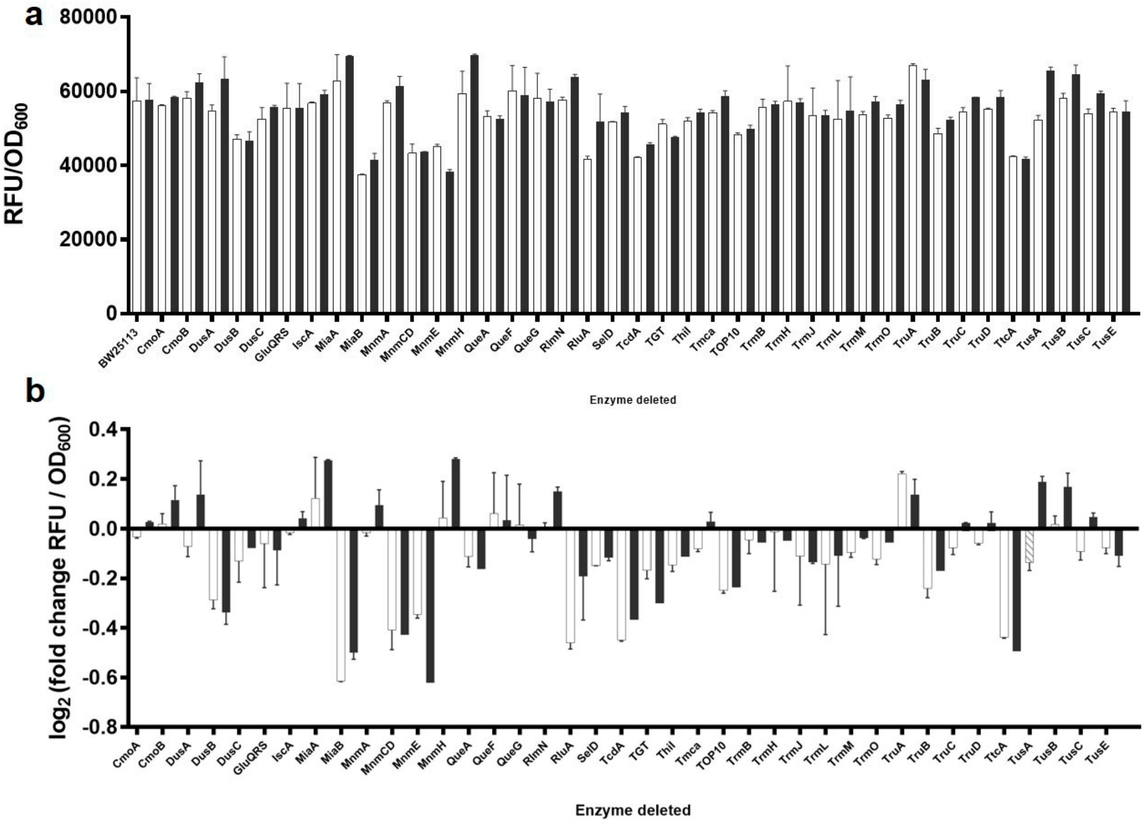

Supplementary Figure S5. Levels of wild type sfGFP synthesis in the tested Keio collection strains expressed in relative fluorescence units (**a**) and as fold change with respect to the synthesis in parental strain BW25113 (**b**). Cells were grown in LB medium without supplementation (empty columns) or with 5 mM Sep added (black columns). Values are mean +/- S.D., N=2-3.

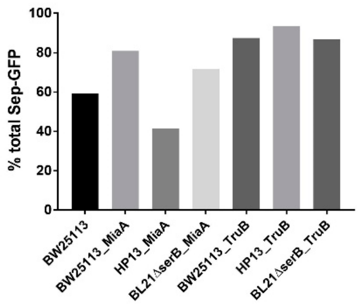

Supplementary Figure S6. Quantification of the shifted bands corresponding to Sep-GFP yields observed in presence of increased amounts of MiaA and TruB (Fig. 5B). Percentages of Sep-modified GFP in the corresponding isolate are shown.

Supplementary Table S1. List of Keio knockout strains employed in this study. Adapted according to MODOMICS database ([1], http://modomics.genesilico.pl).

	Synonym	Full name	RNA substrate	Modification type	Position in tRNA	Substrate base present in $MjtRNA^{Sep}{}_{CUA}{}^{G37A}$
CmoA	YecO	tRNA (uridine-5-oxyacetic acid methyl ester)(34) synthase	tRNA	cmo^{5}U, mcmo^{5}U	34	no
CmoB	YecP	tRNA 5-methoxyuridine(34) synthase	tRNA	cmo^{5}U, mcmo^{5}U	34	no
DusA	YjbN	tRNA dihydrouridine synthase A	tRNA	D	20	
DusB	YhdG	tRNA dihydrouridine synthase B	tRNA	D	16, 17, 20, 20a	yes (U20)
DusC	YohI	tRNA dihydrouridine synthase C	tRNA	D	16, 17, 20, 20a	yes (U20)
GluQRS	YadB	tRNA glutamyl-Q(34) synthetase	tRNA	gluQtRNA	34	no
IscA	YfhF	Iron-binding protein IscA	none	none	none	-
IscS	NuvC	Cysteine desulfurase IscS subfamily	none	none	none	-
MiaA	TrpX	tRNA (adenosine(37)-N6)-dimethylallyltransferase	tRNA	i^{6}A	37	yes
MiaB	YleA	tRNA (N6-isopentenyl adenosine(37)-C2)-methylthiotransferase	tRNA	ms^{2}i^{6}A	37	yes
MnmA	TrmU, AsuE, YcfB	tRNA 2-thiouridine(34) synthase	tRNA	s^{2}U, mnm^{5}s^{2}U	34	no
MnmCD	TrmC, MnmC	tRNA (5-methylaminomethyl-2-thiouridylate)-methyltransferase / FAD-dependent cmnm(5)s(2)U34 oxidoreductase	tRNA	mnm^{5}s^{2}U, mnm^{5}U	34	no

MnmE	ThdF, TrmE	tRNA uridine(34) 5-carboxymethylaminomethyl synthesis GTPase	tRNA	mnm^5s^2U, $cmnm^5s^2U$, $cmnm^5Um$, mnm^5U	34	no
MnmG	GidA, TrmF	tRNA uridine(34) 5-carboxymethylaminomethyl synthesis enzyme	tRNA	mnm^5s^2U, $cmnm^5s^2U$, $cmnm^5Um$, mnm^5U	34	no
MnmH	SelU, YbbB	tRNA 2-selenouridine(34) synthase	tRNA	$cmnm^5ges^2U$, mnm^5se^2U, mnm^5ges^2U	34	no
QueA		tRNA preQ1(34) S-adenosylmethionine ribosyltransferase-isomerase	tRNA	QtRNA	34	no
QueF	YgcD	preQ1 synthase	tRNA	QtRNA	34	no
QueG	YjeS	tRNA epoxyqueuosine(34) reductase	tRNA	QtRNA	34	no
RlmN	TrmG, YfgB	tRNA (adenosine(37)-C2)-methyltransferase / Ribosomal RNA large subunit methyltransferase N	tRNA,rRNA	m^2A	37	yes
RluA	YabO	tRNA pseudouridine(32) synthase / Ribosomal large subunit pseudouridine synthase A	tRNA,rRNA	Y	32	no
SelD	FdhB	Selenide,water dikinase	none	none	none	-
TcdA	YgdL, CsdL	tRNA threonylcarbamoyladenosine dehydratase A	tRNA	ct^6A	37	yes
Tgt	JW0396	tRNA guanosine(34) transglycosylase	tRNA	QtRNA	34	no
ThiI	NuvA	tRNA 4-thiouridine(8) synthase	tRNA	s^4U	8	yes
TmcA	YpfI, JW2459	tRNA cytidine(34) acetyltransferase	tRNA	ac^4C	34	yes
TrmA	RumT	tRNA (uridine(54)-C5)-methyltransferase	tRNA	m^5U	54	yes
TrmB	YggH	tRNA (guanine(46)-N7)-methyltransferase	tRNA	m^7G	46	yes
TrmH	SpoU	tRNA (guanosine(18)-2'-O)-methyltransferase	tRNA	Gm	18	yes
TrmJ	YfhQ, JW2516	tRNA (cytidine(32)/uridine(32)-2'-O)-methyltransferase	tRNA	Um, Cm	32	yes(C32)
TrmL	YibK	tRNA (cytidine(34)-2'-O)-methyltransferase	tRNA	Cm, $cmnm^5Um$	34	yes
TrmM	YfiC, TrmN6	tRNA (adenosine(37)-N6)-methyltransferase	tRNA	m^6A	37	yes
TrmO	TsaA, YaeB	tRNA-methyltransferase O	tRNA	m^6t^6A	37	yes

TruA	HisT, AsuC, HisT, LeuK	tRNA pseudouridine(38,39,40) synthase	tRNA	Y	38, 39, 40	yes(U39)
TruB	YhbA	tRNA pseudouridine(55) synthase	tRNA	Y	55	yes
TruC	YqcB	tRNA pseudouridine(65) synthase	tRNA	Y	65	no
TruD	YgbO	tRNA pseudouridine(13) synthase	tRNA	Y	13	yes
TtcA	YdaO	tRNA 2-thiocytidine(32) synthetase	tRNA	s^2C	32	yes
TusA	sirA, yhhP	tRNA 2-thiouridine(34) synthase	tRNA	none	none	-
TusB	yheL	tRNA 2-thiouridine(34) synthase	tRNA	none	none	-
TusC	yheM	tRNA 2-thiouridine(34) synthase	tRNA	none	none	-
TusD	yheN	tRNA 2-thiouridine(34) synthase	tRNA	none	none	-
TusE	yccK	tRNA 2-thiouridine synthesizing protein E	tRNA	none	none	-

Supplementary Table S2. Plasmids used in this study.

**Name**	**Description**	**Antibiotic resistance**	*** Source**
pET-sfGFP-2TAG -sepT[a]	pET-Duet1 containing the gene encoding sfGFP with S2TAG mutation and *sepT* encoding amber suppressor derived from *Methanocaldococcus jannaschii* $tRNA^{Cys}$	Amp	This study
pET-sfGFP-2TAG	identical to pET-sfGFP-2TAG -sepT but without the sepT gene		
pET-sfGFP	identical to pET-sfGFP-2TAG but with a wild type sfGFP sequence		
pET-sfGFP-2TAG -sepT(G37A)	pET-Duet1 containing the gene encoding sfGFP with S2TAG mutation and *sepT* with a G37A mutation	Amp	This study
pET-sfGFP-2TAG -sepT-Trm5	pET-Duet1 containing the gene encoding sfGFP with S2TAG mutation, *Methanococcus maripaludis* Trm5 gene under lpp promoter and *sepT* encoding amber suppressor derived from *M. jannaschii* $tRNA^{Cys}$		
pET-sfGFP151TAG-sepT$_{G37A}$	pET-Duet1 containing the gene encoding sfGFP with Y151TAG mutation and *sepT* gene with a G37A mutation	Amp	This study
pCDF2-SepRS	pCDF2 containing gene encoding SepRS[b]	Sp	This study

pCDF-lpp.SepRS-Para.MiaA	pCDF containing the gene encoding SepRS under lpp and MiaA under arabinose promoter	Sp	This study
pCDF-lpp.SepRS-Para.TruB	pCDF containing the gene encoding SepRS under lpp and TruB under arabinose promoter	Amp	This study

[a] In all pET-sfGFP constructs, GFP gene was placed under the control of T5, and tRNA (if present) under an lpp promoter

[b]Gene for SepRS was under lpp promoter

Supplementary Table S3. Statistical analysis of the fluorescence measurements obtained for the amber codon suppression efficiency (sfGFP-2TAG synthesis) in the selected Keio deletion strains in presence of Sep-OTS. The fluorescence measurements were calculated as a $\log_2$ fold change relative to the BW25113 strain. The results of the Student's t-test are shown. Theoretical mean value is 0. Supplementation with 5 mM Sep is indicated by "+".

Gene deleted	Mean	Std. Deviation	Std. Error of Mean	Actual mean	Discrepancy	95% CI of discrepancy	t, df	P value (two tailed)	Significant (alpha=0.05)?
cmoA	0.4212	0.01414	0.006325	0.4212	0.4212	0.4036 to 0.4387	t=66.59 df=4	<0.0001	Yes
cmoA +	0.6328	0.06042	0.03488	0.6328	0.6328	0.4828 to 0.7829	t=18.14 df=2	0.003	Yes
cmoB	0.2968	0.2406	0.1076	0.2968	0.2968	-0.001901 to 0.5956	t=2.759 df=4	0.0509	No
cmoB +	0.5878	0.2933	0.1312	0.5878	0.5878	0.2237 to 0.952	t=4.482 df=4	0.011	Yes
dusA	1.025	0.02839	0.01639	1.025	1.025	0.9545 to 1.096	t=62.53 df=2	0.0003	Yes
dusA +	0.8149	0.0113	0.006525	0.8149	0.8149	0.7869 to 0.843	t=124.9 df=2	<0.0001	Yes
dusB	0.5722	0.001129	0.000652	0.5722	0.5722	0.5694 to 0.575	t=877.8 df=2	<0.0001	Yes
dusB +	0.7879	0.04469	0.0258	0.7879	0.7879	0.6769 to 0.8989	t=30.54 df=2	0.0011	Yes
dusC	0.329	0.2333	0.1043	0.329	0.329	0.03931 to 0.6187	t=3.153 df=4	0.0344	Yes
dusC +	0.3225	0.2566	0.1148	0.3225	0.3225	0.003804 to 0.6411	t=2.81 df=4	0.0483	Yes
qluQRS	-0.3944	0.3295	0.1647	-0.3944	-0.3944	-0.9188 to 0.1299	t=2.394 df=3	0.0964	No
gluQRS +	-0.1599	0.3728	0.1864	-0.1599	-0.1599	-0.7532 to 0.4333	t=0.8579 df=3	0.454	No
iscA	1.209	0.3327	0.1664	1.209	1.209	0.6791 to 1.738	t=7.265 df=3	0.0054	Yes
iscA +	1.111	0.4526	0.2263	1.111	1.111	0.3913 to 1.832	t=4.912 df=3	0.0162	Yes
iscS	2.641	0.06597	0.02693	2.641	2.641	2.572 to 2.71	t=98.06 df=5	<0.0001	Yes
iscS +	3.249	0.682	0.2784	3.249	3.249	2.533 to 3.965	t=11.67 df=5	<0.0001	Yes
miAA	-0.3791	0.2525	0.1262	-0.3791	-0.3791	-0.7808 to 0.02259	t=3.003 df=3	0.0575	No
miaA +	-0.3474	0.0793	0.03965	-0.3474	-0.3474	-0.4736 to -0.2213	t=8.762 df=3	0.0031	Yes
miaB	0.8201	0.08743	0.0391	0.8201	0.8201	0.7115 to 0.9286	t=20.97 df=4	<0.0001	Yes
miaB +	0.6187	0.4018	0.1797	0.6187	0.6187	0.1198 to 1.118	t=3.443 df=4	0.0262	Yes
mnmA	-0.09548	0.1624	0.08121	-0.09548	-0.09548	-0.3539 to 0.163	t=1.176 df=3	0.3245	No

Gene deleted	Mean	Std. Deviation	Std. Error of Mean	Actual mean	Discrepancy	95% CI of discrepancy	t, df	P value (two tailed)	Significant (alpha=0.05)?
mnmA +	0.105	0.3079	0.1539	0.105	0.105	-0.3849 to 0.5949	t=0.6823 df=3	0.544	No
mnmCD	1.415	0.04914	0.02457	1.415	1.415	1.337 to 1.494	t=57.6 df=3	<0.0001	Yes
mnmCD +	1.316	0.1308	0.0654	1.316	1.316	1.108 to 1.524	t=20.12 df=3	0.0003	Yes
mnmE	0.8683	0.1062	0.04748	0.8683	0.8683	0.7365 to 1	t=18.29 df=4	<0.0001	Yes
mnmE +	1.887	0.4985	0.2229	1.887	1.887	1.268 to 2.506	t=8.466 df=4	0.0011	Yes
mnmG	0.02361	1.238	0.5536	0.02361	0.02361	-1.513 to 1.561	t=0.04264 df=4	0.968	No
mnmG +	0.7424	1.016	0.4545	0.7424	0.7424	-0.5194 to 2.004	t=1.634 df=4	0.1777	No
mnmH	0.4887	0.01468	0.008477	0.4887	0.4887	0.4522 to 0.5252	t=57.65 df=2	0.0003	Yes
mnmH +	0.6717	0.02601	0.01502	0.6717	0.6717	0.6071 to 0.7363	t=44.73 df=2	0.0005	Yes
queA	1.033	0.2964	0.1482	1.033	1.033	0.5608 to 1.504	t=6.966 df=3	0.0061	Yes
queA +	1.231	0.2062	0.1031	1.231	1.231	0.9033 to 1.56	t=11.94 df=3	0.0013	Yes
queF	0.9549	0.3596	0.1608	0.9549	0.9549	0.5084 to 1.402	t=5.937 df=4	0.004	Yes
queF +	0.3354	1.144	0.5115	0.3354	0.3354	-1.085 to 1.756	t=0.6557 df=4	0.5478	No
queG	0.2985	0.2533	0.1266	0.2985	0.2985	-0.1045 to 0.7015	t=2.357 df=3	0.0996	No
queG +	0.6772	0.2897	0.1449	0.6772	0.6772	0.2162 to 1.138	t=4.675 df=3	0.0185	Yes
rlmN	0.4471	0.4562	0.204	0.4471	0.4471	-0.1193 to 1.014	t=2.192 df=4	0.0935	No
rlmN +	0.874	0.4922	0.2201	0.874	0.874	0.2629 to 1.485	t=3.971 df=4	0.0165	Yes
rluA	0.1163	0.03216	0.01608	0.1163	0.1163	0.06512 to 0.1675	t=7.231 df=3	0.0055	Yes
rluA +	0.2611	0.03212	0.01606	0.2611	0.2611	0.21 to 0.3122	t=16.26 df=3	0.0005	Yes
selD	0.6873	0.2211	0.1106	0.6873	0.6873	0.3354 to 1.039	t=6.217 df=3	0.0084	Yes
selD +	0.7591	0.2774	0.1387	0.7591	0.7591	0.3176 to 1.201	t=5.472 df=3	0.012	Yes
tcdA	0.4659	0.124	0.062	0.4659	0.4659	0.2686 to 0.6632	t=7.514 df=3	0.0049	Yes
tcdA +	0.617	0.08613	0.04307	0.617	0.617	0.48 to 0.7541	t=14.33 df=3	0.0007	Yes
tgt	1.205	0.1109	0.05546	1.205	1.205	1.028 to 1.381	t=21.72 df=3	0.0002	Yes
tgt +	1.309	0.0794	0.0397	1.309	1.309	1.183 to 1.436	t=32.99 df=3	<0.0001	Yes
thiI	-0.1781	0.1869	0.09343	-0.1781	-0.1781	-0.4754 to 0.1193	t=1.906 df=3	0.1527	No
thiI +	-0.04168	0.1574	0.0787	-0.04168	-0.04168	-0.2921 to 0.2088	t=0.5296 df=3	0.6331	No
tmcA	0.1532	0.04364	0.02182	0.1532	0.1532	0.08379 to 0.2227	t=7.022 df=3	0.0059	Yes
tmcA +	0.3442	0.05134	0.02567	0.3442	0.3442	0.2625 to 0.4259	t=13.41 df=3	0.0009	Yes
TOP10	0.6162	0.173	0.0865	0.6162	0.6162	0.3409 to 0.8915	t=7.124 df=3	0.0057	Yes
TOP10 +	0.625	0.23	0.115	0.625	0.625	0.259 to 0.9911	t=5.434 df=3	0.0122	Yes

Gene deleted	Mean	Std. Deviation	Std. Error of Mean	Actual mean	Discrepancy	95% CI of discrepancy	t, df	P value (two tailed)	Significant (alpha=0.05)?
trmA	0.2308	0.5573	0.2786	0.2308	0.2308	-0.656 to 1.117	t=0.8282 df=3	0.4683	No
trmA +	0.393	0.5327	0.2663	0.393	0.393	-0.4546 to 1.241	t=1.476 df=3	0.2365	No
trmB	-0.07429	0.724	0.362	-0.07429	-0.07429	-1.226 to 1.078	t=0.2052 df=3	0.8505	No
trmB +	0.1082	0.7623	0.3811	0.1082	0.1082	-1.105 to 1.321	t=0.284 df=3	0.7949	No
trmH	0.6263	0.1095	0.04895	0.6263	0.6263	0.4904 to 0.7622	t=12.79 df=4	0.0002	Yes
trmH +	0.5318	0.3477	0.1555	0.5318	0.5318	0.1 to 0.9635	t=3.42 df=4	0.0268	Yes
trmJ	0.1963	0.3267	0.1633	0.1963	0.1963	-0.3235 to 0.7161	t=1.202 df=3	0.3156	No
trmJ +	0.5501	0.08019	0.04009	0.5501	0.5501	0.4225 to 0.6777	t=13.72 df=3	0.0008	Yes
trmL	0.1738	0.3256	0.1628	0.1738	0.1738	-0.3443 to 0.692	t=1.068 df=3	0.364	No
trmL +	0.2891	0.3565	0.1783	0.2891	0.2891	-0.2782 to 0.8564	t=1.622 df=3	0.2033	No
trmM	0.4305	0.06643	0.02971	0.4305	0.4305	0.348 to 0.513	t=14.49 df=4	0.0001	Yes
trmM +	0.656	0.1057	0.04729	0.656	0.656	0.5246 to 0.7873	t=13.87 df=4	0.0002	Yes
trmO	0.2074	0.2415	0.108	0.2074	0.2074	-0.09253 to 0.5073	t=1.92 df=4	0.1273	No
trmO +	0.5309	0.1116	0.04993	0.5309	0.5309	0.3923 to 0.6695	t=10.63 df=4	0.0004	Yes
truA	2.108	0.4166	0.1473	2.108	2.108	1.759 to 2.456	t=14.31 df=7	<0.0001	Yes
truA +	2.333	0.3483	0.1232	2.333	2.333	2.042 to 2.625	t=18.95 df=7	<0.0001	Yes
truB	-0.562	0.1208	0.0604	-0.562	-0.562	-0.7542 to -0.3698	t=9.305 df=3	0.0026	Yes
truB +	-0.2878	0.1469	0.06571	-0.2878	-0.2878	-0.4702 to -0.1053	t=4.379 df=4	0.0119	Yes
truC	0.46	0.04539	0.02269	0.46	0.46	0.3878 to 0.5322	t=20.27 df=3	0.0003	Yes
truC +	0.6966	0.1795	0.08974	0.6966	0.6966	0.411 to 0.9823	t=7.763 df=3	0.0044	Yes
truD	0.1721	0.3759	0.1681	0.1721	0.1721	-0.2946 to 0.6388	t=1.024 df=4	0.3638	No
truD +	0.5062	0.2874	0.1285	0.5062	0.5062	0.1494 to 0.863	t=3.939 df=4	0.017	Yes
ttcA	0.7246	0.4769	0.2133	0.7246	0.7246	0.1324 to 1.317	t=3.397 df=4	0.0274	Yes
ttcA +	0.6751	0.7312	0.327	0.6751	0.6751	-0.2327 to 1.583	t=2.065 df=4	0.1079	No
tusA	0.4458	0.524	0.2139	0.4458	0.4458	-0.104 to 0.9957	t=2.084 df=5	0.0916	No
tusA +	1.394	0.2742	0.1119	1.394	1.394	1.107 to 1.682	t=12.46 df=5	<0.0001	Yes
tusB	0.7278	1.057	0.3997	0.7278	0.7278	-0.2502 to 1.706	t=1.821 df=6	0.1185	No
tusB +	2.039	0.3713	0.1516	2.039	2.039	1.649 to 2.429	t=13.45 df=5	<0.0001	Yes
tusC	0.6023	0.05913	0.02957	0.6023	0.6023	0.5082 to 0.6964	t=20.37 df=3	0.0003	Yes
tusC +	0.859	0.1088	0.05439	0.859	0.859	0.6859 to 1.032	t=15.79 df=3	0.0006	Yes
tusD	1.015	0.04516	0.02019	1.015	1.015	0.9588 to 1.071	t=50.26 df=4	<0.0001	Yes

Gene deleted	Mean	Std. Deviation	Std. Error of Mean	Actual mean	Discrepancy	95% CI of discrepancy	t, df	P value (two tailed)	Significant (alpha=0.05)?
tusD +	1.12	0.05405	0.02417	1.12	1.12	1.053 to 1.187	t=46.32 df=4	<0.0001	Yes
tusE	0.08858	0.1851	0.09257	0.08858	0.08858	-0.206 to 0.3832	t=0.9568 df=3	0.4092	No
tusE +	0.2664	0.2414	0.1207	0.2664	0.2664	-0.1178 to 0.6506	t=2.207 df=3	0.1144	No

Supplementary Table S4. Statistical analysis of the fluorescence measurements obtained for the amber codon suppression efficiency (sfGFP-2TAG synthesis) in the selected Keio deletion strains in presence of $tRNA^{Sep}$ only. The fluorescence measurements were calculated as a log_2 fold change relative to the BW25113 strain. The results of the Student's t-test are shown. Theoretical mean value is 0. Supplementation with 5 mM Sep is indicated by "+".

Gene deleted	Mean	Std. Deviation	Std. Error of Mean	Actual mean	Discrepancy	95% CI of discrepancy	t, df	P value (two tailed)	Significant (alpha=0.05)?
cmoA	0.0906	0.698	0.349	0.0906	0.0906	-1.02 to 1.201	t=0.2596 df=3	0.812	No
cmoA +	0.1184	0.6709	0.3354	0.1184	0.1184	-0.9491 to 1.186	t=0.3529 df=3	0.7475	No
cmoB	0.4771	0.5895	0.2948	0.4771	0.4771	-0.4609 to 1.415	t=1.619 df=3	0.2039	No
cmoB +	0.5727	0.562	0.281	0.5727	0.5727	-0.3215 to 1.467	t=2.038 df=3	0.1343	No
dusA	0.1713	0.2544	0.1272	0.1713	0.1713	-0.2336 to 0.5762	t=1.347 df=3	0.2708	No
dusA +	-0.2667	0.1154	0.05769	-0.2667	-0.2667	-0.4503 to -0.0831	t=4.623 df=3	0.0191	Yes
dusB	0.5075	0.128	0.064	0.5075	0.5075	0.3038 to 0.7112	t=7.93 df=3	0.0042	Yes
dusB +	0.5607	0.2821	0.1411	0.5607	0.5607	0.1118 to 1.01	t=3.975 df=3	0.0285	Yes
dusC	0.124	0.3548	0.1774	0.124	0.124	-0.4406 to 0.6885	t=0.6989 df=3	0.5349	No
dusC +	-0.03174	0.341	0.1705	-0.03174	-0.03174	-0.5744 to 0.5109	t=0.1862 df=3	0.8642	No
gluQRS	-0.3779	0.0566	0.03268	-0.3779	-0.3779	-0.5185 to -0.2373	t=11.57 df=2	0.0074	Yes
gluQRS +	-0.4843	0.09237	0.05333	-0.4843	-0.4843	-0.7137 to -0.2548	t=9.081 df=2	0.0119	Yes
iscA	0.8254	0.04885	0.0282	0.8254	0.8254	0.7041 to 0.9468	t=29.27 df=2	0.0012	Yes
iscA +	0.7044	0.05809	0.03354	0.7044	0.7044	0.5601 to 0.8487	t=21 df=2	0.0023	Yes
iscS	0.9832	1.504	0.7519	0.9832	0.9832	-1.41 to 3.376	t=1.308 df=3	0.2822	No
iscS +	0.7606	1.625	0.8123	0.7606	0.7606	-1.825 to 3.346	t=0.9363 df=3	0.4182	No
miAA	0.1147	0.4237	0.173	0.1147	0.1147	-0.3299 to 0.5594	t=0.6634 df=5	0.5364	No
miaA +	0.1088	0.2987	0.1219	0.1088	0.1088	-0.2046 to 0.4223	t=0.8925 df=5	0.413	No
miaB	0.3088	0.01587	0.009162	0.3088	0.3088	0.2693 to 0.3482	t=33.7 df=2	0.0009	Yes
miaB +	0.2149	0.01236	0.007138	0.2149	0.2149	0.1842 to 0.2456	t=30.1 df=2	0.0011	Yes
mnmA	0.2491	0.8941	0.4471	0.2491	0.2491	-1.174 to 1.672	t=0.5572 df=3	0.6163	No
mnmA +	0.1474	0.8971	0.4485	0.1474	0.1474	-1.28 to 1.575	t=0.3287 df=3	0.764	No
mnmCD	1.134	0.1347	0.06733	1.134	1.134	0.92 to 1.349	t=16.85 df=3	0.0005	Yes
mnmCD +	1.013	0.04154	0.02077	1.013	1.013	0.9466 to 1.079	t=48.76 df=3	<0.0001	Yes
mnmE	0.672	0.4086	0.1827	0.672	0.672	0.1647 to 1.179	t=3.678 df=4	0.0212	Yes
mnmE +	1.014	0.8384	0.3749	1.014	1.014	-0.02653 to 2.056	t=2.706 df=4	0.0538	No
mnmG	0.2405	0.1924	0.1111	0.2405	0.2405	-0.2375 to 0.7185	t=2.164 df=2	0.1629	No
mnmG +	0.2252	0.1695	0.09787	0.2252	0.2252	-0.1959 to 0.6463	t=2.301 df=2	0.1481	No
mnmH	0.2885	0.3093	0.1547	0.2885	0.2885	-0.2038 to 0.7807	t=1.865 df=3	0.159	No

Gene deleted	Mean	Std. Deviation	Std. Error of Mean	Actual mean	Discrepancy	95% CI of discrepancy	t, df	P value (two tailed)	Significant (alpha=0.05)?
mnmH +	0.2525	0.1761	0.08803	0.2525	0.2525	-0.02769 to 0.5326	t=2.868 df=3	0.0642	No
queA	0.8851	0.08516	0.04258	0.8851	0.8851	0.7496 to 1.021	t=20.79 df=3	0.0002	Yes
queA +	1.096	0.1821	0.09106	1.096	1.096	0.8066 to 1.386	t=12.04 df=3	0.0012	Yes
queF	0.176	0.1473	0.07363	0.176	0.176	-0.05831 to 0.4103	t=2.391 df=3	0.0967	No
queF +	0.05339	0.1429	0.07146	0.05339	0.05339	-0.174 to 0.2808	t=0.7471 df=3	0.5092	No
queG	0.3834	0.5552	0.2776	0.3834	0.3834	-0.5001 to 1.267	t=1.381 df=3	0.2612	No
queG +	0.252	0.4748	0.2374	0.252	0.252	-0.5035 to 1.007	t=1.061 df=3	0.3664	No
rlmN	0.4231	0.3082	0.1541	0.4231	0.4231	-0.06726 to 0.9134	t=2.746 df=3	0.071	No
rlmN +	0.2313	0.3535	0.1767	0.2313	0.2313	-0.3311 to 0.7937	t=1.309 df=3	0.2818	No
rluA	0.5111	0.3028	0.1514	0.5111	0.5111	0.0292 to 0.9929	t=3.375 df=3	0.0432	Yes
rluA +	0.4956	0.2493	0.1246	0.4956	0.4956	0.09891 to 0.8922	t=3.976 df=3	0.0285	Yes
selD	0.3499	0.2883	0.1442	0.3499	0.3499	-0.1089 to 0.8087	t=2.427 df=3	0.0936	No
selD +	0.2951	0.2266	0.1133	0.2951	0.2951	-0.0655 to 0.6558	t=2.604 df=3	0.0801	No
tcdA	-0.9499	1.73	0.7063	-0.9499	-0.9499	-2.766 to 0.8657	t=1.345 df=5	0.2364	No
tcdA +	-0.2518	0.5033	0.2055	-0.2518	-0.2518	-0.78 to 0.2764	t=1.225 df=5	0.275	No
tgt	0.2598	0.7691	0.3846	0.2598	0.2598	-0.964 to 1.484	t=0.6756 df=3	0.5477	No
tgt +	0.06248	0.3862	0.1931	0.06248	0.06248	-0.552 to 0.677	t=0.3236 df=3	0.7675	No
thiI	-0.2298	0.1638	0.08189	-0.2298	-0.2298	-0.4904 to 0.03076	t=2.807 df=3	0.0675	No
thiI +	0.3367	0.5895	0.2948	0.3367	0.3367	-0.6013 to 1.275	t=1.142 df=3	0.3362	No
tmcA	0.6546	0.04383	0.02531	0.6546	0.6546	0.5457 to 0.7635	t=25.87 df=2	0.0015	Yes
tmcA +	0.5745	0.02093	0.01208	0.5745	0.5745	0.5225 to 0.6265	t=47.55 df=2	0.0004	Yes
TOP10	1.241	0.009848	0.005686	1.241	1.241	1.216 to 1.265	t=218.2 df=2	<0.0001	Yes
TOP10 +	1.18	0.01977	0.01142	1.18	1.18	1.131 to 1.229	t=103.4 df=2	<0.0001	Yes
trmA	0.3005	0.0975	0.04875	0.3005	0.3005	0.1454 to 0.4557	t=6.165 df=3	0.0086	Yes
trmA +	0.5086	0.5625	0.2812	0.5086	0.5086	-0.3864 to 1.404	t=1.808 df=3	0.1683	No
trmB	-0.1125	0.5475	0.2738	-0.1125	-0.1125	-0.9838 to 0.7587	t=0.411 df=3	0.7087	No
trmB +	-0.1175	0.6008	0.3004	-0.1175	-0.1175	-1.073 to 0.8384	t=0.3912 df=3	0.7218	No
trmH	0.5643	0.2134	0.1067	0.5643	0.5643	0.2247 to 0.904	t=5.288 df=3	0.0132	Yes
trmH +	0.4695	0.07603	0.03802	0.4695	0.4695	0.3485 to 0.5904	t=12.35 df=3	0.0011	Yes
trmJ	-0.6247	0.4524	0.2262	-0.6247	-0.6247	-1.345 to 0.09519	t=2.762 df=3	0.0701	No
trmJ +	-0.6937	0.4639	0.2319	-0.6937	-0.6937	-1.432 to 0.04449	t=2.991 df=3	0.0581	No
trmL	0.5893	0.04341	0.0217	0.5893	0.5893	0.5203 to 0.6584	t=27.15 df=3	0.0001	Yes
trmL +	0.6431	0.07241	0.03621	0.6431	0.6431	0.5279 to 0.7583	t=17.76 df=3	0.0004	Yes
trmM	0.3675	0.2686	0.1343	0.3675	0.3675	-0.05989 to 0.7949	t=2.737 df=3	0.0715	No
trmM +	0.3262	0.2504	0.1252	0.3262	0.3262	-0.07226 to 0.7247	t=2.605 df=3	0.08	No
trmO	0.4556	0.3113	0.1557	0.4556	0.4556	-0.03981 to 0.9509	t=2.927 df=3	0.0612	No
trmO +	0.3205	0.2749	0.1374	0.3205	0.3205	-0.1168 to 0.7579	t=2.332 df=3	0.1019	No
truA	2.304	0.251	0.1255	2.304	2.304	1.905 to 2.703	t=18.36 df=3	0.0004	Yes
truA +	1.912	0.09473	0.04736	1.912	1.912	1.762 to 2.063	t=40.38 df=3	<0.0001	Yes
truB	-0.1864	0.1698	0.0849	-0.1864	-0.1864	-0.4566 to 0.08379	t=2.196 df=3	0.1157	No
truB +	-0.2649	0.1201	0.06006	-0.2649	-0.2649	-0.456 to -0.07374	t=4.41 df=3	0.0216	Yes

Gene deleted	Mean	Std. Deviation	Std. Error of Mean	Actual mean	Discrepancy	95% CI of discrepancy	t, df	P value (two tailed)	Significant (alpha=0.05)?
truC	0.2865	0.04022	0.02011	0.2865	0.2865	0.2225 to 0.3505	t=14.25 df=3	0.0007	Yes
truC +	0.2881	0.09082	0.04541	0.2881	0.2881	0.1436 to 0.4326	t=6.344 df=3	0.0079	Yes
truD	-0.09493	0.1901	0.09505	-0.09493	-0.09493	-0.3974 to 0.2076	t=0.9987 df=3	0.3915	No
truD +	-0.05004	0.1037	0.05186	-0.05004	-0.05004	-0.2151 to 0.115	t=0.9651 df=3	0.4057	No
ttcA	-0.2315	0.05636	0.03254	-0.2315	-0.2315	-0.3715 to -0.09152	t=7.115 df=2	0.0192	Yes
ttcA +	-0.1381	0.3297	0.1903	-0.1381	-0.1381	-0.957 to 0.6809	t=0.7253 df=2	0.5436	No
tusA	0.692	0.07203	0.03601	0.692	0.692	0.5774 to 0.8066	t=19.21 df=3	0.0003	Yes
tusA +	0.9288	0.2984	0.1492	0.9288	0.9288	0.4541 to 1.404	t=6.226 df=3	0.0084	Yes
tusB	0.7939	0.1534	0.07668	0.7939	0.7939	0.5499 to 1.038	t=10.35 df=3	0.0019	Yes
tusB +	0.858	0.2492	0.1246	0.858	0.858	0.4614 to 1.255	t=6.884 df=3	0.0063	Yes
tusC	0.3857	0.3307	0.1653	0.3857	0.3857	-0.1405 to 0.9119	t=2.333 df=3	0.1019	No
tusC +	0.239	0.2618	0.1309	0.239	0.239	-0.1776 to 0.6556	t=1.826 df=3	0.1654	No
tusD	0.8727	0.02977	0.01719	0.8727	0.8727	0.7987 to 0.9466	t=50.77 df=2	0.0004	Yes
tusD +	0.7785	0.03836	0.02215	0.7785	0.7785	0.6832 to 0.8738	t=35.15 df=2	0.0008	Yes
tusE	-0.7681	0.04562	0.02281	-0.7681	-0.7681	-0.8407 to -0.6955	t=33.67 df=3	<0.0001	Yes
tusE +	-0.8365	0.1193	0.05967	-0.8365	-0.8365	-1.026 to -0.6466	t=14.02 df=3	0.0008	Yes

Supplementary Table S5. Statistical analysis of the fluorescence measurements obtained for the wild type sfGFP synthesis in the selected Keio deletion strains in presence of Sep-OTS. The fluorescence measurements were calculated as a log_2 fold change relative to the BW25113 strain. The results of the Student's t-test are shown. Theoretical mean value is 0. Supplementation with 5 mM Sep is indicated by "+".

Gene deleted	Mean	Std. Deviation	Std. Error of Mean	Actual mean	Discrepancy	95% CI of discrepancy	t, df	P value (two tailed)	Significant (alpha=0.05)?
CmoA	-0.03225	0.005784	0.00409	-0.03225	-0.03225	-0.08422 to 0.01972	t=7.885 df=1	0.0803	No
CmoA+Sep	0.02755	0.002376	0.00168	0.02755	0.02755	0.006207 to 0.0489	t=16.4 df=1	0.0388	Yes
CmoB	0.01941	0.04072	0.02879	0.01941	0.01941	-0.3464 to 0.3852	t=0.6742 df=1	0.6224	No
CmoB+Sep	0.1157	0.05793	0.04096	0.1157	0.1157	-0.4048 to 0.6362	t=2.824 df=1	0.2167	No
DusA	-0.07001	0.04225	0.02988	-0.07001	-0.07001	-0.4496 to 0.3096	t=2.343 df=1	0.2568	No
DusA+Sep	0.1366	0.1367	0.09664	0.1366	0.1366	-1.091 to 1.364	t=1.413 df=1	0.392	No
DusB	-0.2868	0.03661	0.02589	-0.2868	-0.2868	-0.6157 to 0.04206	t=11.08 df=1	0.0573	No
DusB+Sep	-0.3054	0.08038	0.05684	-0.3054	-0.3054	-1.028 to 0.4168	t=5.374 df=1	0.1171	No
DusC	-0.1306	0.08521	0.06025	-0.1306	-0.1306	-0.8961 to 0.635	t=2.167 df=1	0.2753	No
DusC+Sep	-0.04587	0.01509	0.01067	-0.04587	-0.04587	-0.1814 to 0.08971	t=4.299 df=1	0.1455	No
GluQRS	-0.05919	0.1785	0.1262	-0.05919	-0.05919	-1.663 to 1.545	t=0.4689 df=1	0.7209	No
GluQRS+Sep	-0.05466	0.1717	0.1214	-0.05466	-0.05466	-1.597 to 1.488	t=0.4502 df=1	0.7307	No
IscA	-0.01573	0.00787	0.005565	-0.01573	-0.01573	-0.08644 to 0.05499	t=2.826 df=1	0.2165	No
IscA+Sep	0.04171	0.0268	0.01895	0.04171	0.04171	-0.1991 to 0.2825	t=2.201 df=1	0.2715	No
MiaA	0.123	0.1643	0.1162	0.123	0.123	-1.353 to 1.599	t=1.059 df=1	0.4818	No
MiaA+Sep	0.2757	0.002455	0.001736	0.2757	0.2757	0.2536 to 0.2977	t=158.8 df=1	0.004	Yes
MiaB	-0.6134	0.002878	0.002035	-0.6134	-0.6134	-0.6393 to -0.5876	t=301.4 df=1	0.0021	Yes

Gene deleted	Mean	Std. Deviation	Std. Error of Mean	Actual mean	Discrepancy	95% CI of discrepancy	t, df	P value (two tailed)	Significant (alpha=0.05)?
MiaB+Sep	-0.4675	0.05858	0.04143	-0.4675	-0.4675	-0.9938 to 0.05889	t=11.28 df=1	0.0563	No
MnmA	-0.01481	0.01512	0.01069	-0.01481	-0.01481	-0.1506 to 0.121	t=1.385 df=1	0.398	No
MnmA+Sep	0.09416	0.06264	0.04429	0.09416	0.09416	-0.4686 to 0.6569	t=2.126 df=1	0.2799	No
MnmCD	-0.4076	0.08048	0.05691	-0.4076	-0.4076	-1.131 to 0.3155	t=7.162 df=1	0.0883	No
MnmCD+Sep	-0.3945	0.000743	0.000525	-0.3945	-0.3945	-0.4012 to -0.3879	t=751.5 df=1	0.0008	Yes
MnmE	-0.3455	0.01546	0.008928	-0.3455	-0.3455	-0.3839 to -0.3071	t=38.7 df=2	0.0007	Yes
MnmE+Sep	-0.5877	0.02386	0.01687	-0.5877	-0.5877	-0.8021 to -0.3733	t=34.84 df=1	0.0183	Yes
MnmH	0.04349	0.1469	0.1039	0.04349	0.04349	-1.277 to 1.364	t=0.4186 df=1	0.7476	No
MnmH+Sep	0.2796	0.006213	0.004393	0.2796	0.2796	0.2238 to 0.3354	t=63.65 df=1	0.01	Yes
QueA	-0.1117	0.04272	0.03021	-0.1117	-0.1117	-0.4955 to 0.2721	t=3.699 df=1	0.1681	No
QueA+Sep	-0.1301	0.02525	0.01786	-0.1301	-0.1301	-0.3569 to 0.09681	t=7.284 df=1	0.0869	No
QueF	0.0614	0.164	0.1159	0.0614	0.0614	-1.412 to 1.535	t=0.5296 df=1	0.6899	No
QueF+Sep	0.03325	0.1822	0.1288	0.03325	0.03325	-1.603 to 1.67	t=0.2582 df=1	0.8392	No
QueG	0.01521	0.1641	0.116	0.01521	0.01521	-1.459 to 1.49	t=0.1311 df=1	0.917	No
QueG+Sep	-0.00789	0.08583	0.06069	-0.00789	-0.00789	-0.779 to 0.7632	t=0.13 df=1	0.9177	No
RlmN	0.004665	0.01914	0.01353	0.004665	0.004665	-0.1673 to 0.1766	t=0.3446 df=1	0.7887	No
RlmN+Sep	0.1499	0.01796	0.0127	0.1499	0.1499	-0.01147 to 0.3113	t=11.8 df=1	0.0538	No
RluA	-0.4601	0.02504	0.01771	-0.4601	-0.4601	-0.6851 to -0.2352	t=25.99 df=1	0.0245	Yes
RluA+Sep	-0.1594	0.2086	0.1043	-0.1594	-0.1594	-0.4914 to 0.1725	t=1.529 df=3	0.2238	No
SelD	-0.1485	0.001089	0.00077	-0.1485	-0.1485	-0.1583 to -0.1387	t=192.9 df=1	0.0033	Yes
SelD+Sep	-0.08434	0.04494	0.03178	-0.08434	-0.08434	-0.4881 to 0.3195	t=2.654 df=1	0.2294	No
TcdA	-0.4478	0.006392	0.00452	-0.4478	-0.4478	-0.5053 to -0.3904	t=99.08 df=1	0.0064	Yes
TcdA+Sep	-0.3353	0.01882	0.01331	-0.3353	-0.3353	-0.5044 to -0.1662	t=25.19 df=1	0.0253	Yes
TGT	-0.1667	0.03516	0.02487	-0.1667	-0.1667	-0.4827 to 0.1492	t=6.705 df=1	0.0942	No
TGT+Sep	-0.2674	0.004964	0.00351	-0.2674	-0.2674	-0.312 to -0.2228	t=76.18 df=1	0.0084	Yes
ThiI	-0.1453	0.02666	0.01885	-0.1453	-0.1453	-0.3848 to 0.09421	t=7.708 df=1	0.0821	No
ThiI+Sep	-0.07967	0.02105	0.01489	-0.07967	-0.07967	-0.2688 to 0.1095	t=5.352 df=1	0.1176	No
Tmca	-0.0804	0.01222	0.00864	-0.0804	-0.0804	-0.1902 to 0.02938	t=9.306 df=1	0.0682	No
Tmca+Sep	0.02813	0.03782	0.02675	0.02813	0.02813	-0.3117 to 0.368	t=1.052 df=1	0.4839	No
TOP10	-0.248	0.01224	0.006119	-0.248	-0.248	-0.2675 to -0.2285	t=40.53 df=3	<0.0001	Yes
TOP10+Sep	-0.2041	0.02981	0.01491	-0.2041	-0.2041	-0.2515 to -0.1566	t=13.69 df=3	0.0008	Yes
TrmB	-0.04462	0.05638	0.03987	-0.04462	-0.04462	-0.5512 to 0.462	t=1.119 df=1	0.4642	No
TrmB+Sep	-0.02259	0.02017	0.01427	-0.02259	-0.02259	-0.2038 to 0.1587	t=1.583 df=1	0.3586	No
TrmH	-0.01267	0.2396	0.1694	-0.01267	-0.01267	-2.165 to 2.14	t=0.07479 df=1	0.9525	No
TrmH+Sep	-0.01482	0.02867	0.02027	-0.01482	-0.01482	-0.2724 to 0.2428	t=0.7309 df=1	0.5982	No
TrmJ	-0.1095	0.1995	0.1411	-0.1095	-0.1095	-1.902 to 1.683	t=0.7759 df=1	0.5799	No
TrmJ+Sep	-0.1026	0.03742	0.02646	-0.1026	-0.1026	-0.4388 to 0.2336	t=3.878 df=1	0.1607	No
TrmL	-0.1421	0.2854	0.2018	-0.1421	-0.1421	-2.706 to 2.422	t=0.7043 df=1	0.6094	No
TrmL+Sep	-0.07548	0.2376	0.168	-0.07548	-0.07548	-2.211 to 2.06	t=0.4492 df=1	0.7312	No
TrmM	-0.09532	0.02042	0.01444	-0.09532	-0.09532	-0.2788 to 0.08816	t=6.601 df=1	0.0957	No
TrmM+Sep	-0.0052	0.03484	0.02463	-0.0052	-0.0052	-0.3182 to 0.3078	t=0.211 df=1	0.8676	No

Gene deleted	Mean	Std. Deviation	Std. Error of Mean	Actual mean	Discrepancy	95% CI of discrepancy	t, df	P value (two tailed)	Significant (alpha=0.05)?
TrmO	-0.1219	0.02289	0.01619	-0.1219	-0.1219	-0.3276 to 0.0837	t=7.534 df=1	0.084	No
TrmO+Sep	-0.02313	0.02652	0.01875	-0.02313	-0.02313	-0.2614 to 0.2151	t=1.234 df=1	0.4337	No
TruA	0.2209	0.009734	0.006883	0.2209	0.2209	0.1335 to 0.3084	t=32.1 df=1	0.0198	Yes
TruA+Sep	0.1364	0.06268	0.04432	0.1364	0.1364	-0.4267 to 0.6996	t=3.078 df=1	0.2	No
TruB	-0.2392	0.03931	0.0278	-0.2392	-0.2392	-0.5923 to 0.114	t=8.605 df=1	0.0737	No
TruB+Sep	-0.1366	0.02193	0.01551	-0.1366	-0.1366	-0.3336 to 0.06038	t=8.812 df=1	0.0719	No
TruC	-0.07562	0.02865	0.02026	-0.07562	-0.07562	-0.333 to 0.1818	t=3.732 df=1	0.1666	No
TruC+Sep	0.02293	0.001341	0.000949	0.02293	0.02293	0.01088 to 0.03498	t=24.18 df=1	0.0263	Yes
TruD	-0.05717	0.007729	0.005465	-0.05717	-0.05717	-0.1266 to 0.01227	t=10.46 df=1	0.0607	No
TruD+Sep	0.02265	0.04479	0.03167	0.02265	0.02265	-0.3798 to 0.4251	t=0.7152 df=1	0.6047	No
TtcA	-0.4377	0.004059	0.00287	-0.4377	-0.4377	-0.4742 to -0.4013	t=152.5 df=1	0.0042	Yes
TtcA+Sep	-0.4615	0.01819	0.01286	-0.4615	-0.4615	-0.6249 to -0.2981	t=35.89 df=1	0.0177	Yes
TusA	-0.1359	0.03326	0.02352	-0.1359	-0.1359	-0.4348 to 0.1629	t=5.778 df=1	0.1091	No
TusA+Sep	0.1893	0.02198	0.01554	0.1893	0.1893	-0.008112 to 0.3868	t=12.18 df=1	0.0521	No
TusB	0.01747	0.03315	0.02344	0.01747	0.01747	-0.2804 to 0.3153	t=0.7453 df=1	0.5922	No
TusB+Sep	0.1687	0.05544	0.0392	0.1687	0.1687	-0.3294 to 0.6668	t=4.303 df=1	0.1454	No
TusC	-0.09136	0.03504	0.02478	-0.09136	-0.09136	-0.4062 to 0.2234	t=3.687 df=1	0.1686	No
TusC+Sep	0.04732	0.01534	0.01085	0.04732	0.04732	-0.09052 to 0.1852	t=4.362 df=1	0.1435	No
TusE	-0.07592	0.0249	0.01437	-0.07592	-0.07592	-0.1378 to -0.01408	t=5.282 df=2	0.034	Yes
TusE+Sep	-0.07581	0.07636	0.04409	-0.07581	-0.07581	-0.2655 to 0.1139	t=1.72 df=2	0.2276	No

Supplementary references

1. Boccaletto, P.; Machnicka, M.A.; Purta, E.; Piatkowski, P.; Baginski, B.; Wirecki, T.K.; de Crecy-Lagard, V.; Ross, R.; Limbach, P.A.; Kotter, A., *et al.* MODOMICS: a database of RNA modification pathways. 2017 update. *Nucleic Acids Res* **2018**, *46*, D303-D307.
